# Loop 7 of E2 Enzymes: An Ancestral Conserved Functional Motif Involved in the E2-Mediated Steps of the Ubiquitination Cascade

**DOI:** 10.1371/journal.pone.0040786

**Published:** 2012-07-18

**Authors:** Elena Papaleo, Nicola Casiraghi, Alberto Arrigoni, Marco Vanoni, Paola Coccetti, Luca De Gioia

**Affiliations:** Department of Biotechnology and Biosciences, University of Milano-Bicocca, Milan, Italy; King’s College, United Kingdom

## Abstract

The ubiquitin (Ub) system controls almost every aspect of eukaryotic cell biology. Protein ubiquitination depends on the sequential action of three classes of enzymes (E1, E2 and E3). E2 Ub-conjugating enzymes have a central role in the ubiquitination pathway, interacting with both E1 and E3, and influencing the ultimate fate of the substrates. Several E2s are characterized by an extended acidic insertion in loop 7 (L7), which if mutated is known to impair the proper E2-related functions. In the present contribution, we show that acidic loop is a conserved ancestral motif in E2s, relying on the presence of alternate hydrophobic and acidic residues. Moreover, the dynamic properties of a subset of family 3 E2s, as well as their binary and ternary complexes with Ub and the cognate E3, have been investigated. Here we provide a model of L7 role in the different steps of the ubiquitination cascade of family 3 E2s. The L7 hydrophobic residues turned out to be the main determinant for the stabilization of the E2 inactive conformations by a tight network of interactions in the catalytic cleft. Moreover, phosphorylation is known from previous studies to promote E2 competent conformations for Ub charging, inducing electrostatic repulsion and acting on the L7 acidic residues. Here we show that these active conformations are stabilized by a network of hydrophobic interactions between L7 and L4, the latter being a conserved interface for E3-recruitment in several E2s. In the successive steps, L7 conserved acidic residues also provide an interaction interface for both Ub and the Rbx1 RING subdomain of the cognate E3. Our data therefore suggest a crucial role for L7 of family 3 E2s in all the E2-mediated steps of the ubiquitination cascade. Its different functions are exploited thank to its conserved hydrophobic and acidic residues in a finely orchestrate mechanism.

## Introduction

The ubiquitin (Ub) system controls almost every aspect of eukaryotic cell biology, finely coordinating and tuning the amplitude and duration of cellular signal by the modulation of protein-protein interactions and the targeting of selected proteins for proteasomal degradation [1]–[3]. Protein ubiquitination of target substrates relies on the sequential action of three classes of enzymes, the E1 Ub-activating enzyme, E2 Ub-conjugating enzyme and the E3 ligase [4]. Moreover, ubiquitin-like (Ubl) proteins have been identified, as SUMO or Nedd8 [4]–[6]. Even if signals mediated by mono-ubiquitination of selected substrates have been recurrently identified, polyubiquitin (polyUb) chains are the modifications that more frequently mediate a broad array of diverse cellular signals and functions [7]. The topology of polyUb chains ultimately dictates the achieved effects and depends on the different Ub lysines residues (K11, K48 or K63) involved in the cross-linking of the Ub molecules [3], [8]–[12]. Defects in the Ub or Ubl pathways are associated with several diseases, from cancer to neurodegenerative disorders [13]–[15]. Moreover, the enzymes of the Ubl cascade very recently turned out to be promising therapeutic targets [16], [17], thanks to their modular nature and the notion that they can be modulated or inhibited [18]–[20]. However, the mechanisms involved in each step of the ubiquitination process and the interactions between the different enzymes of the cascade are still not well understood in their molecular details, with the exception of some recent studies focusing on the last steps in the cascade, which require the action of E3 enzymes [21]–[24].

**Figure 1:**
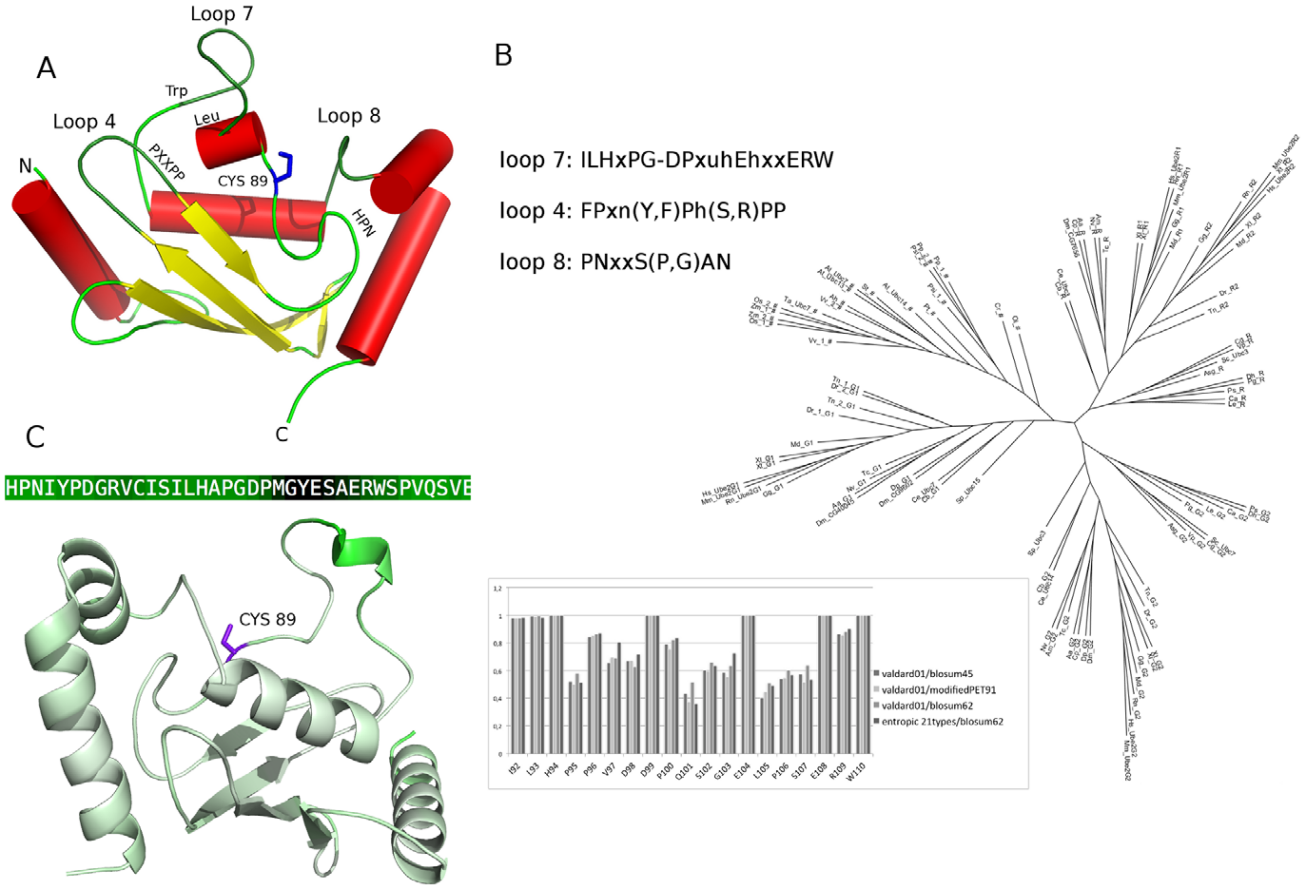
E2 UBC domain and phylogenetic tree of E2 family 3. **A**) The 3D structure of the E2 UBC domain is shown, along with the conserved residues in the E2 superfamily. The loops L7, L4 and L8, the catalytic cysteine, the invariant Trp, the HPN (His-Pro-Asn) motif, as well as the proline-rich motif (PPxxP) are indicated as reported in (26). The structure has been adapted by the 3D X-ray structure of Ube2g2 (PDB code 2CYX). **B**) The unrooted phylogenetic tree was obtained by PHYLIP package from multiple sequence alignments of E2 family 3 sequences, reported in Table S1. For each protein a label referred to the different sub-families (R, R1, R2, G1, G2 and #) is reported. In the lower-left box the conservation degree of each residues of L7 loop is indicated according to different scoring function. The consensus pattern for L4, L7 and L8 are also reported. **C**) The 3D structure of Ube2g2 is used as a reference and each residue coloured with different shade of green according to Cα rmsf values calculated from the MD simulations (from light green to dark green for increasing rmsf values). The rmsf intensity of each residue of L7 is indicated on the primary sequence of L7 in the upper box. The data from MD simulations of each E2 enzymes of family 3 considered in this study are reported in Text S1.

Instead, E2 Ub-conjugating enzymes reside at the heart of the ubiquitination pathway, interacting both with E1 and E3 enzymes, and are key mediators of protein ubiquitination, Ub chain assembly and topology [25]–[29]. Nevertheless, a full understanding of the molecular mechanisms related to E2 activity and interactions with cognate E1, Ub or Ubl, and E3s, is still far from being achieved. The yeast and human genome encodes more than 10/40 E2s, allowing for a multitude of distinct ubiquitination events [4], [30]. Several classification by phylogenetic analyses [31], [32] and sequence/structure similarity have been proposed [32], [33]. In particular, E2s have been recently classified in 17 families, by phylogenetic analysis of seven genomes [31]. All E2s share a conserved catalytic core domain (Ub-conjugating domain, UBC), which is the minimum sufficient unit for E2 activity and adopts a α/β fold (Figure 1A). The UBC domain contains the catalytic cysteine and the interaction interfaces for E1 and E3 enzymes, along with conserved sequence motifs (Figure 1A). The active-site cysteine (Figure 1A) is located in a shallow cleft interacting with the Ub C-terminal tail [34]. Despite the conservation of the UBC domain fold, many E2 proteins contain sequence insertion or extension of the 150 residue core region. Among these, enzymes belonging to family 3 of E2s, also known as Cdc34-like E2s, are characterized by a flexible and disordered 12/13-residues insertion in the β4α2 loop (loop 7, L7), the so-called “acidic loop” (103–112, Ube2g numbering), downstream from the active site cysteine [31]. The acidic loop has been demonstrated relevant for proper catalytic function and polyubiquitination [35]–[37], and its conformation is known to be regulated by phosphorylation of distal conserved serines in the UBC domain [38], [39]. Beyond the essential role of the acidic residues in the L7 insertion, few details are known about its structural properties and how it promotes catalytic function, as well as its role in the different steps of ubiquitination.

In light of the above scenario, we carried out a computational study on a subset of representative members of family 3 E2s. Particular attention was devoted to the L7-mediated intra- and inter-molecular interactions and different computational approaches were employed, from sequence and phylogenetic analyses of 100 sequences of E2 family 3 enzymes, coarse grained calculations and all-atom multi-replica molecular dynamics (MD) simulations (overall collecting more than 3 µs of MD trajectories).

## Results

### The L7 Acidic Loop of E2 Family 3 is an Invariant Ancestral Motif Characterized by Alternate Hydrophobic and Acidic Residues

Multiple sequence and structural alignments were carried out, comparing more than 100 representative members of E2 family 3 (E2-f3) enzymes, isolated from 40 different organisms (Table S1, Figure S1). The 12/13-amino acid acidic loop insertions downstream the catalytic cysteine is present only in family 3, whereas other families (families 5, 7 and 14) are characterized by less extended insertions of few residues [31]. Phylogenetic analysis of E2-f3 members allow to identify four different sub-families (namely R, G1, G2 and #, according to [31]), in agreement with previous analyses carried out on a smaller subset of 20 sequences derived from 7 organisms [31] (Figure 1B). Our analysis of a higher number of family 3 sequences, which widely span the tree of life, allows to better clarify specific characteristics of E2-f3 members and also to catch differences among the sub-families.

Indeed, it turns out that all the E2-f3 enzymes are characterized by the 12/13 amino acid insertion in L7, in which we identified a pattern of strongly conserved alternate hydrophobic and acidic residues (Figure S1; Figure 1B). In particular, the pattern of hydrophobic/acidic residues in L7 insertion is an invariant feature of all the family 3 members, independently of the sub-family (Figure 1B, Figure S1). The conservation degree of L7 residues (Figure 1B, lower panel) was estimated, as well as a common consensus pattern of the whole L7 loop (residues 92–110 or 97–114 in Ube2g2 and yeast Ubc3 (Cdc34), respectively) was defined, *ILHxPG-DPxuhEhxxERW*, where *x* indicates non conserved amino acids, *h* hydrophobic residues and *u* tiny residues (A, G or S). I92, L93, P96, G97, P99 are strictly conserved, as well as hydrophobic residues at positions 101/102 and 104 (Ube2g2 numbering). Three acidic residues (D98, E103 and E107, Ube2g2 numbering) in L7 also emerge as invariant across all the family 3 members and spatially separated by hydrophobic residues.

The strong conservation of both amino acid composition and length of the acidic loop strongly suggests that the division in sub-families was an evolutionary event that took place after the appearance of the acidic insertion in L7. In fact, it was previously pointed out that the subdivision of family 3 in different sub-families was also anterior to the separation between the *Animalia* and *Fungi* phyla, which has been estimated around 1.3 billion years ago [31]. Therefore, our analyses identify an ancestral and conserved structural and sequence motif in E2 enzymes. To enforce this notion, we also found E2 sequences ascribable to family 3 and conserving the acidic/hydrophobic insertion in the loop in some bacterial species, indicating the ancient origin of these E2 enzymes.

The invariant Trp residue (W110 in Ube2g2) is located few residues downstream the 12/13-residues insertion. X-ray structures of the complexes between E2 enzymes and RING or HECT E3 proteins reveal that W110 is located close to a proline (P112 in Ube2g2) at the tip of L7, as well as to the two prolines at the base of L4 [40]. The latter belong to a proline-rich motif (Y/FPxxPP) 7/11 amino acids upstream to the HPN motif [31]. According to our multiple sequence alignment, the pro-rich motif in L4 of family 3 can be summarized by the consensus pattern *FPxn(Y,F)Ph(S,R)PP*, where *x* indicates non conserved amino acids, *h* hydrophobic residues and *n* acidic residues. Interactions between W110 and the prolines were speculated to stabilize the L7 loop and contribute to the correct reciprocal orientation of L4 and L7 loop for E3 recognition mediated by L4 specific residues [31]. In the proximity of the catalytic cleft also loop 8 (L8) is located, which is also rich of hydrophobic residues and is characterized by a consensus sequence *PNxxS(P,G)AN* in family 3 (Figure S1, Figure 1B).

The role of the L7 acidic residues, with particular attention to the effects induced by their mutations on protein function, was thoroughly characterized by biochemical assays [35]–[37]. Nevertheless, a rationale of the effects at the molecular level is still missing or at least incomplete, as well as no information is available about the L7 hydrophobic residues. The high conservation of a group of hydrophobic residues in L7, derived from our alignments and phylogenetic investigation, prompted us to further investigate their role and interactions using a multiscale simulation approach.

### A Network of Chained Correlated Motions Involving Hydrophobic Residues of the Acidic Loop 7 and Loop 8 during Native Protein Dynamics

At first, the methods available in FlexServ [41], Normal Mode Analysis (NMA), Brownian Dynamics (BD) and Discrete Molecular Dynamics (DMD), have been carried out on 10 three-dimensional (3D) structures of E2-f3, both using the experimentally known 3D structures and a subset of homology models. In particular, we include in the study the X-ray structures of *Saccharomyces cerevisiae* Ubc7 (pdb entry 2UCZ), human Ube2g2 (pdb entry 2CYX), *Caenorhabditis elegans* Ubc7 (pdb entry 1PZV), *Mus musculus* Ube2g2 (pdb entry 3FSH), the NMR resolution of human Ube2g2 (pdb entry 2KLY), the average structures from the MD simulations of yeast Cdc34 (Ubc3) [39]. Furthermore, homology models were carried out from *Saccharomyces pombe* Ubc3 (SpUbc3), *Arabidopsis thaliana* Ubc7 (AtUbc7) *and Drosophila melanogaster* (Dm_CG40045 E2).

In particular, from the dynamic ensemble collected by each of the methods mentioned above, chained correlations were calculated by post-processing of the dynamical cross-correlation matrix (DCCM) of atomic fluctuations (see Materials and Methods). The analysis of networks of correlated residues is useful to determine the connections between movements of different protein residues and their communication across the 3D structure [21], [24], [42]–[44]. In several cases, these coupled residues, besides defining sequence connections, allow the identification of biologically relevant intramolecular “communication” pathway [41]. In particular, chained correlations may highlight residues which are characterized by long-range communication through the calculation of intermediate correlations.

**Figure 2:**
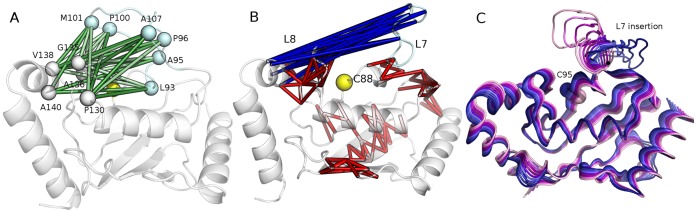
Intramolecular communication between hydrophobic residues of L7 and L8 and conformational variability of L7 in the native state. **A**) Chained correlations between L7 hydrophobic residues (coloured in cyan) and L8 residues are indicated by sticks of different shades of green related to the depth level at which the long-range correlation has been identified (from depth level 3 (dark green) to 5 (light green)). The secondary structures are indicated as cartoon and the catalytic cysteine highlighted in yellow. A consensus of chained correlations identified for Ube2g2 (pdb entry 2KLZ) are reported as example, whereas the data for each proteins are reported in Table S2. **B**) Anti-correlated motions of L7 and L4 in the native conformational ensemble of E2 family 3 enzymes. The cross-correlated motions in E2 family 3 MD (correlation threshold of 0.35 and time-window for the calculation of 4 ns) are shown as red (positive correlations) and blue (negative correlations, anti-correlated motions) sticks. The catalytic cysteine and L7 residues are shown in yellow and pale-cyan, respectively. **C**) The projections of the first principal component derived by PCA analysis of the MD ensemble on the 3D structure are indicated with different shade of colours for blue to pink.

In this context, we selected as root residues for chain correlations the hydrophobic residues located in the acidic loop and its neighborhoods for each of the available structures. Interestingly, most of L7 hydrophobic residues feature high correlations not only with the residues in their immediate proximity, but also with some distal residues, which are mainly located in L8 on the opposite side of the catalytic cleft with respect to L7. In particular, chained correlations and consequent intramolecular communications during dynamics between a cluster of L7 residue (P96, P100 and M101) and a group of L8 residues (V138, P130, G/P135, Ube2g2 numbering) have been identified (Figure 2A, Table S2).

### Closed Inactive L7 Conformations in the Native Ensemble are Triggered by Coupled Motions between L7 and L8

To explore L7 conformational changes and intra-molecular interactions in atomic details, classical molecular dynamics (MD) simulations of Ubc7, Ubc3 (Cdc34) and Ube2g2 (Table 1) were carried out. In fact, previous investigation pointed out an intrinsic high flexibility of the acidic loop. This was supported by different methods as the crystallographic B-factor of human and yeast Ubc7 [45], [46], recent NMR studies of free Ube2g2 in solution [47] or Ube2g2 in complex with a fragment of its E3 partner [19], as well as root mean square fluctuations from MD simulations of non-phosphorylated and phosphorylated Cdc34 [39], suggesting the possibility of conformational changes in L7. It is not surprisingly therefore that the B-factor on the first 10 principal modes derived by NMA, BD and DMD sampling (Text S1), as well as the Carbon-alpha (Cα) root mean square fluctuations (rmsf) profiles from MD simulations (Figure 1C, Text S1) reveal a high flexibility of the acidic loop, with particular regard to the 12/13 residues insertion in all the E2-f3 here investigated.

**Table 1:** Summary of the multi-replica all-atom MD simulations.

Protein system	Number of*replicas*	Duration per*replica*	Total duration	Starting structure
Cdc34 (Ubc3)	20	20/80 ns	0.92 µs	Cdc34 models using *Ce*Ubc7 (replicas 1–4), hUbe2g2 (replicas 5–8 and 13–20) *Sc*Ubc7 (replicas 9–12) as templates
Cdc34-pS130-pS167	11	50 ns	0.55 µs	Average structure from ensembles A (*replica*s 1–3), B (*replica*s 4–5) and C (*replica*s 6–7), D (*replica*s 8–9), and E (*replica*s 10–11) of native Cdc34 MD [39]
Cdc34-pS130	10	50 ns	0.50 µs	Average structure from ensembles A (*replica*s 1–2), B (*replica*s 3–4) and C (*replica*s 5–6), D (*replica*s 7–8), and E (*replica*s 9–10) of native Cdc34 MD [39]
Ubc7	4	40 ns	0.16 µs	X-ray structure of *Sc*Ubc7 (pdb entry 2UCZ)
Ube2g2_xray_	4	40 ns	0.16 µs	X-ray structure of hUbe2g2 (pdb entry 2CYX)
Ube2g2_NMR_	8	40 ns	0.32 µs	NMR structure of hUbe2g2 (pdb entry 2KLY)
Cdc34-Ub	4	40 ns	0.16 µs	Cdc34-Ub models using as template Ubc1-Ub (pdb entry 1FXT)
Ubc1-Ub	1	40 ns	0.04 µs	Ubc1-Ub NMR structure (pdb entry 1FXT)
Ub-Cdc34-E3	2	40 ns	0.08 µs	Ub-Cdc34-Rbx1 models using as template the UbcH7-cClb RING (pdb entry 1FBV) and Rbx1 (pdb entry 3DQV)

Indeed, the MD simulations here presented allow to better sample the conformational space accessible to L7 in family 3, highlighting larger structural rearrangements and a propensity for exploring mainly closed conformations (with respect to the catalytic cleft) around the native state and in absence of post-translational modifications. In fact, the analyses of residues characterized by coupled motions during MD dynamics, as derived by DCCM analysis (Materials and Methods), point outs in all the simulated E2s several anti-correlated motions between L7 and L8 and α3 helix, respectively (Figure 2B). The anti-correlations indicate the intrinsic tendency of the two regions to approach each other in the native ensemble. This notion is also confirmed by the projection, on the 3D structure, of the first principal component from essential dynamics, which describes the loop motion (Figure 2C).

The coupled motions have also been evaluated considering their time-evolution during each independent replica, to better disclose the relationship between L7 and L8, monitoring DCCM with 1ns and 0.5 ns time-windows. Highly anti-correlated motions in the first part of the trajectories, whose starting structures were open conformations, are evident. They are an index of the propensity of L7 and L8 to approach each other, in agreement with the results from chained correlations described in Figure 2A. Moreover, when L7 and L8 are at a distance lower than 0.6 nm, the anticorrelated motions tend to decrease and further to disappear, in agreement with the established interaction between the two loops and the consequent appearance of correlated motions between them.

The distances between the catalytic site and L7 as a function of catalytic cysteine solvent accessibility, in agreement with this scenario, identify the presence of two main conformational states. Most of the structures collected during native dynamics are characterized by closed or semi-closed L7 conformations, which are indicated by an average distance of less than 0.6 nm between L7 and the catalytic cysteine, along with a solvent-buried Cys, featuring solvent accessibility lower than 10%, as previously observed for Cdc34 [39]. The other population is characterized by semi-open or open L7 conformations with distances higher than 1.0 nm from the catalytic site and a higher accessibility of the catalytic cysteine with solvent accessibility higher than 35%. To better quantify the different subpopulations, the conformational ensemble achieved by the simulations was divided, according to the distance between the acidic residue and the catalytic site in closed (distance lower than 0.6 nm), semi-open (distance between 0.6 and 1.0 nm) and open (distance higher than 1.0 nm) conformations. The classification was also checked according to accessibility of the catalytic cysteine side-chain (<20%, 20%<Cys accessibility<35%, Cys accessibility >35% for closed, semi-open and open E2 conformations, respectively). It turns out that the ensemble is averagely composed by 65%, 9% and 26% of closed, semi-open and open states respectively, according to the distances between acidic loop and the catalytic site. The values are similar if the division is carried out according to solvent accessibility of the catalytic cysteine, collecting 75%, 7% and 18% for closed, semi-open and open states, respectively.

**Figure 3:**
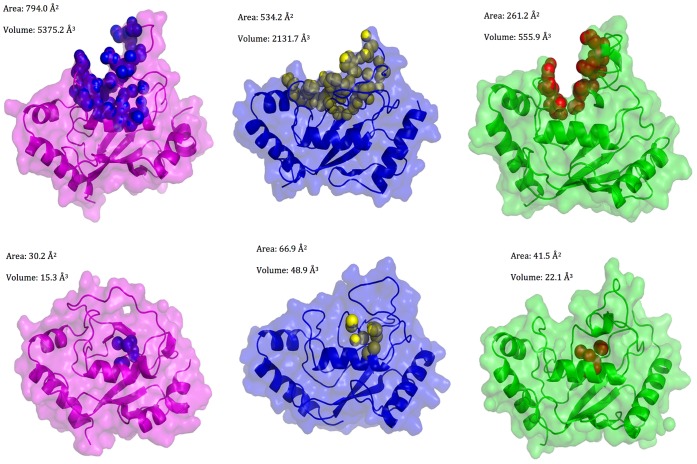
A cavity competent for ubiquitin-charging in E2 “open” conformations. The residues in the solvent-accessible pocket around the catalytic cysteine as calculated by CASTp on representative snapshots from the MD simulations of Ube2g2 (left), Ubc7 (middle) and Cdc34 (right) are indicated by spheres, along with the average area and volume of the cleft in open (upper panels) and closed (lower panels) conformations of L7.

Moreover, calculations of putative pockets and cavities by Cast-P [48] on snapshots from the MD ensemble identify a binding cavity including the catalytic site only in the E2s structures in which L7 is characterized by open conformations (Figure 3). The cavity has an average area of 324,49 Å^2^ in the structures with L7 open conformations, whereas it decreases below 60 Å^2^ or even less in the so-called closed conformations, enforcing the relevance of L7 conformational changes to make the catalytic site accessible for Ub binding.

Our results suggest a general role for L7 as a lid modulating Ub-charging activity in all the E2-f3 members. Moreover, here we showed that, in the inactive non-phosphorylated state, all the Cdc34-like enzymes are more likely to populate conformational states in which L7 is in closed conformations and a buried catalytic site. The driving force for this closure it is to be searched in the conserved hydrophobic residues, previously not characterized and coupled motions during dynamics between L7 and L8 residues.

### Conserved Network of Hydrophobic Interaction between L7 and L8 Stabilized Closed Conformations of E2 Enzymes of Family 3

**Figure 4:**
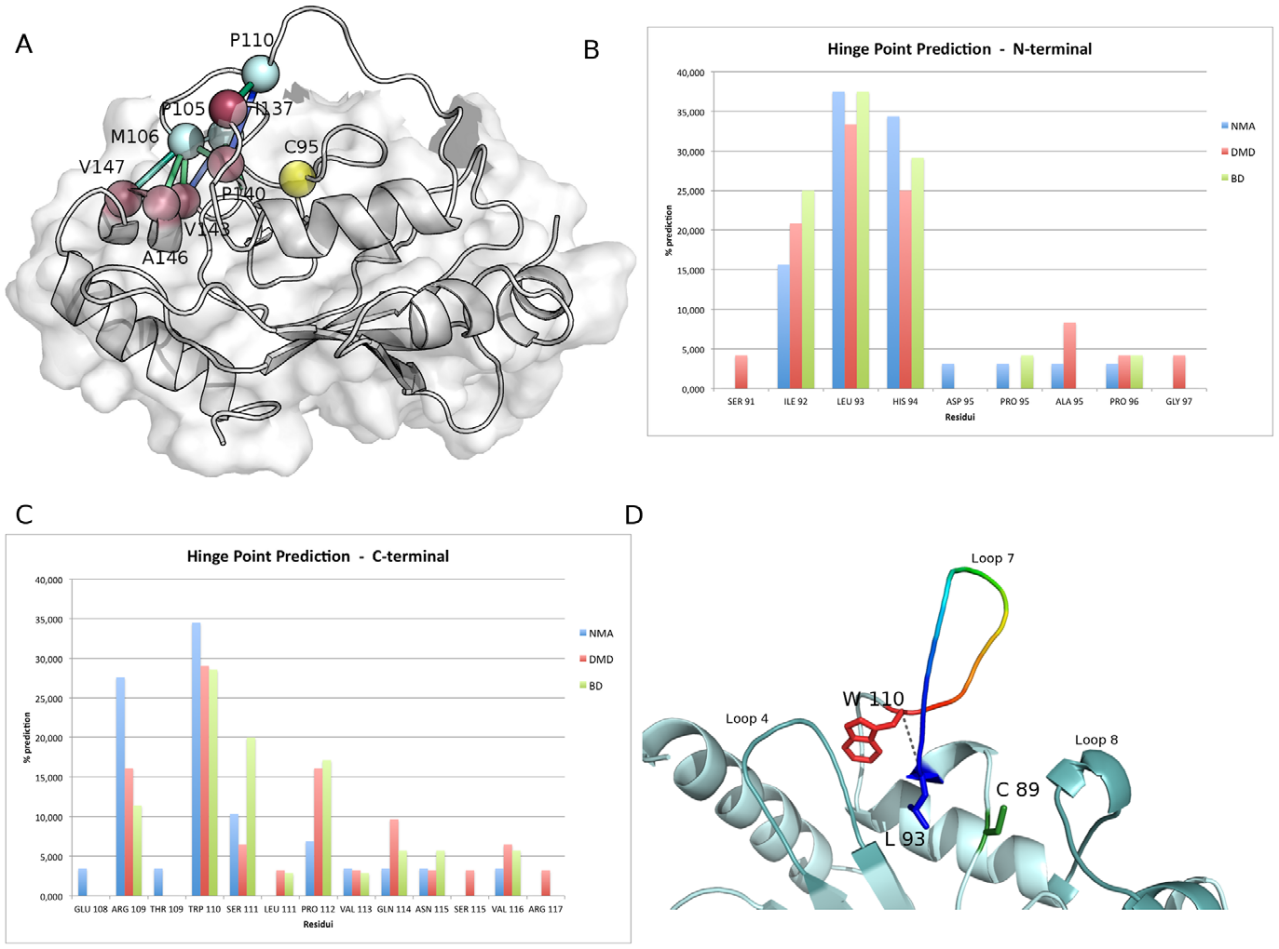
A cluster of hydrophobic interactions between L7 and L8 in inactive E2 conformations and hinges of L7 omega loop. **A**) The networks of hydrophobic interactions between L7 and L8 residues have been monitored in the closed states from MD simulations of Ubc7, Ube2g2 and Cdc34 and their persistence in the ensemble is indicated by sticks coloured from green (low persistence) to blue (high persistence). For sake of clarity, the hydrophobic networks have been mapped on an MD average closed structure of Cdc34 with the catalytic cysteine, the L7 and L8 hydrophobic residues indicated as yellow, light cyan and dark red, respectively. **B–C**) The prediction of L7 hinge points, in all the 11 structures of E2 family 3 members are reported both for the N-terminal (B) and C-terminal hinges (C) calculated by NMA, BD and DMD methods. **D**) The surrounding of the catalytic site of E2 family 3 enzymes is shown, using Ube2g2 as representative members. L7 is coloured according to the hinge prediction values and the identified hinge W110 and L93, along with the average distance between them during the simulation is indicated.

The existence of a tight network of chained cross-correlations between hydrophobic residues in L7 and hydrophobic residues in L8 (Figure 2A, Table S2), which forms one of the side of the catalytic pocket, suggest that hydrophobic interactions are likely to take place between L7 residues and the hydrophobic residues surrounding the catalytic site. Therefore, we selected structures in which L7 is in closed conformations, i.e. all the structures in which the distance between the center of mass of the catalytic cysteine and the center of mass of the acidic loop is lower than 0.6 nm for each E2 macro-trajectory. Furthermore, these “closed E2” ensembles were used to monitor the interactions between hydrophobic residues of L7 and L8 and to calculate their persistence. It turns out a cluster of hydrophobic interactions between L7 and L8 that are stably interconnected during dynamics and promote the E2 “closed” conformations (Figure 4A). A pivotal role emerges for M106, P105 and P110 of L7, which provide a tight network of hydrophobic interactions with P140 and I137 of L8 and A146 and V147 on the adjacent α3 helix (Cdc34 numbering). Hydrophobic interactions among the homologous corresponding residues of L7 and L8 have been also identified in all the other simulated E2-f3 enzymes, pointing out, one more time, a common mechanism mediated by the same conserved residues.

### Loop 7 of E2 Family 3 Classifies as an Omega Loop, which is Generally a Structure Associate to Crucial Regulatory Function in Protein System

Loop 7 of E2-f3 is a long and mostly disordered segment. Random coil structures are known to often play a crucial role in the regulation of biological processes [49]. In several cases, these structures are not merely disordered regions, but adopt a loop-shaped conformation, with a small distance between their extremities (hinges points). The main chain of these segments, connected to the rest of the protein structure by the hinges, traces a conformation resembling a Greek omega, which have been therefore referred as omega loop and they are generally associated with regulatory functions [50]. In this context, considering the strict conservation of the acidic loop in family 3 and its capability to modulate the ubiquitin-charging activity [39], we also assessed if L7 can be classified not merely as an unstructured random coil motif, but as an omega loop, according to parameters proposed in the literature for omega loop classification.

An omega loop is generally defined by its length, the maximum distance between Cα-Cα atoms in the loop which is restrained to well-specific values, the absence of secondary structures apart from few turns, and the distance between the hinges [51]. In fact, the hinges distance should be in the range of 3.7–10 Å, as well as shorter than two-thirds of the longest Cα-Cα distance across the omega segment [51]. All these aspects have been analyzed on the members of E2-f3, using the structures from the MD ensembles. L7, in agreement with omega loop features, includes around 13 residues and it does not contain regular elements of secondary structures, both in the experimental depositions and in the MD conformations. Only in some E2 structures a short 3.10 helix is observed, but it is not considered as a stable secondary structural element and it has been also reported in other omega loops [51].

In order to further verify if L7 can be described as an omega loop, an hinge point prediction has been carried out both analyzing rmsf profiles from single MD *replica*s and macro-trajectories of Ube2g2, Ubc3 and Ubc7 (Text S1), as well as using data from NMA, BD and DMD sampling for all the experimental and model E2 structures previously described (Figure 4B–4C). In fact, a suitable hinge structure should display a fixed domain characterized by low RMSF/B-factor values and a floppy domain characterized by high RMSF/B-factor values. Hinge residues are generally located at the region of sharp slope change between the fixed and floppy domains [52]. At the L7 N-terminal extremity, three putative hinges have been detected, which are also strictly conserved in the family 3 (Figure 1B); I92, L93 and H94 (Ube2g2 numbering) (Figure 4B). All the predictions indeed agree in the identification of the invariant Trp (W110, Ube2g2 numbering) as the C-terminal hinge (Text S1; Figure 4C). The pairwise distances between the Trp Cα atom and each of the 3 putative N-terminal hinges have been monitored in the available MD macro-trajectories to verify if they fall within the accessible range for omega loop [3.7–10 Å]. It turns out a range of distances of 4.2–12 Å, 3.8–10.1 Å and 4.4–12.5 Å for the W110-L92, W110-L93 and W110-H94 pairs, respectively. L93 therefore turns out to be the most suitable N-terminal hinge (Figure 4B–D). The identified hinge points also satisfy the hydrophobic nature suggested by Ring et al. [52]. Moreover, for each system, the maximum distances between Cα-Cα atoms in the loop has been monitored, which turns out to be always shorter than 2/3 of the maximum distance, in line with the requirement to be classified as an omega loop. It is also worth to mention that L7 is also rich in glycine, proline, aspartate, tyrosine, serine and asparagines, which have been suggested to be the most frequent residues in omega loops [51] [49], [53].

### In the Active Phospho-E2s, L7 Open Conformations are Stabilized by Hydrophobic Interactions with L4, as well as by Electrostatic Interactions of L7 Acidic Residues and the Bound Ub

Phosphorylation of Cdc34 catalytic domain was demonstrated to activate Ub-charging activity [38], [39]. In particular, phosphorylation of a conserved serine at position 130 causes electrostatic repulsion with the acidic residues in L7, promoting an outward displacement of the loop and an open and competent conformation of the catalytic cleft for Ub-charging [39].

**Figure 5:**
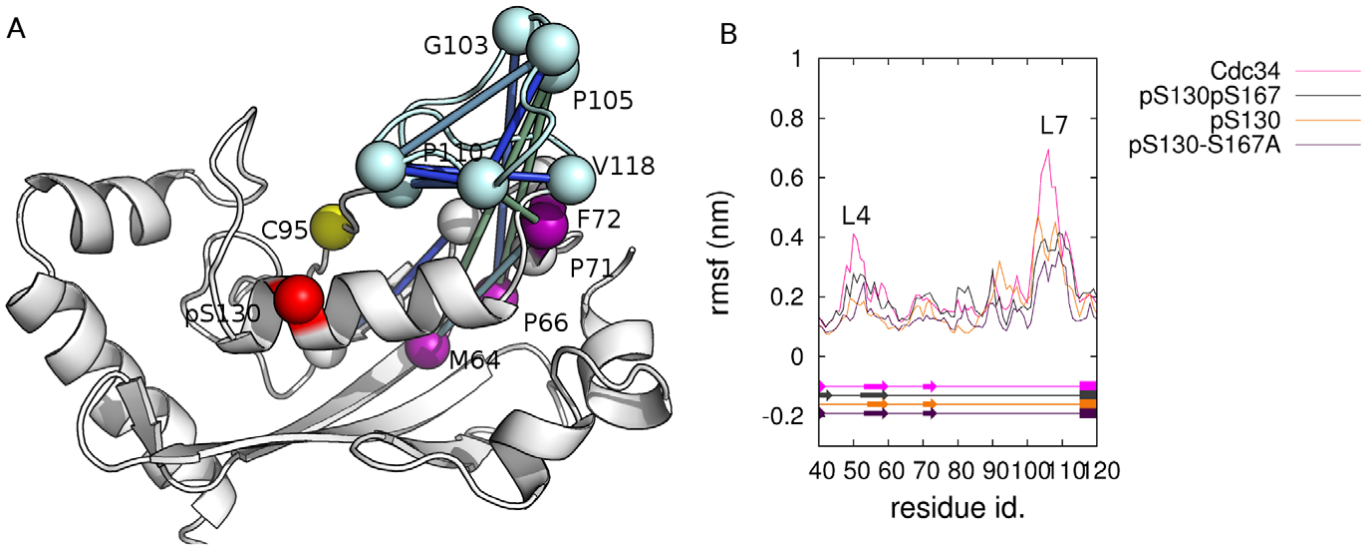
Intermolecular interactions involving the acidic loop in phospho-E2 and in the E2 complexes with ubiquitin or the cognate E3. **A**) The networks of hydrophobic interactions between L7 and L4 residues have been monitored in the open states from MD simulations of phospho-Cdc34 (both pS130-pS167 and pS130 variants) and their persistence in the ensemble is indicated by sticks coloured from green (low persistence) to blue (high persistence). For sake of clarity, the hydrophobic networks have been mapped on an average open structure of Cdc34 with the catalytic cysteine, the L7 and L4 hydrophobic residues indicated as yellow, light cyan and magenta, respectively. **B**) Cα rmsf profiles from phospho- and non-phosphorylated Cdc34 variants. The rmsf peaks relative to L7 insertion and L4 are indicated by the arrow.

In light of the above scenario, we analyzed the simulations of phospho-Cdc34 variants with particular attention to the L7 hydrophobic residues, which were not considered in the previous study and in the available literature as far as we know. Since the phospho-enzyme is stabilized in an open conformation, the L7 hydrophobic residues are no longer able to interact with residues of the catalytic pocket. It turns out that when the acidic loop is stabilized in the open conformation by phosphorylation, its hydrophobic residues rearrange to interact with hydrophobic conserved residues in L4 (Figure 1B, Figure 5A). In particular, the interactions involve some residues of the proline-rich motif, which are conserved in the whole E2 superfamily [31]. The phosphorylation not only stabilized L7, as previously observed, reducing its atomic fluctuation but also L4, indicating that the hydrophobic interactions between L7 and L4 in phospho-variants of E2-f3 enzymes are implicated in the reciprocal stabilization of the conformation of these two loops. In fact, a concomitant decrease of atomic fluctuations of L7 and L4 can be identified in the comparison between rmsf profiles of phospho- and wild-type Cdc34 variants (Figure 5B), which also well fit with strongly correlated motions (higher, in absolute value, than 0.4) between the two loops induced by phosphorylation. These correlations are absent ore very weak (lower, in absolute value, than 0.2) in the not phosphorylated variant.

The L7 open conformation allows, in turn, a full solvent-accessible orientation of its acidic residues (side-chain solvent accessible surface, SAS >70%) in absence of the Ub molecule.

**Figure 6:**
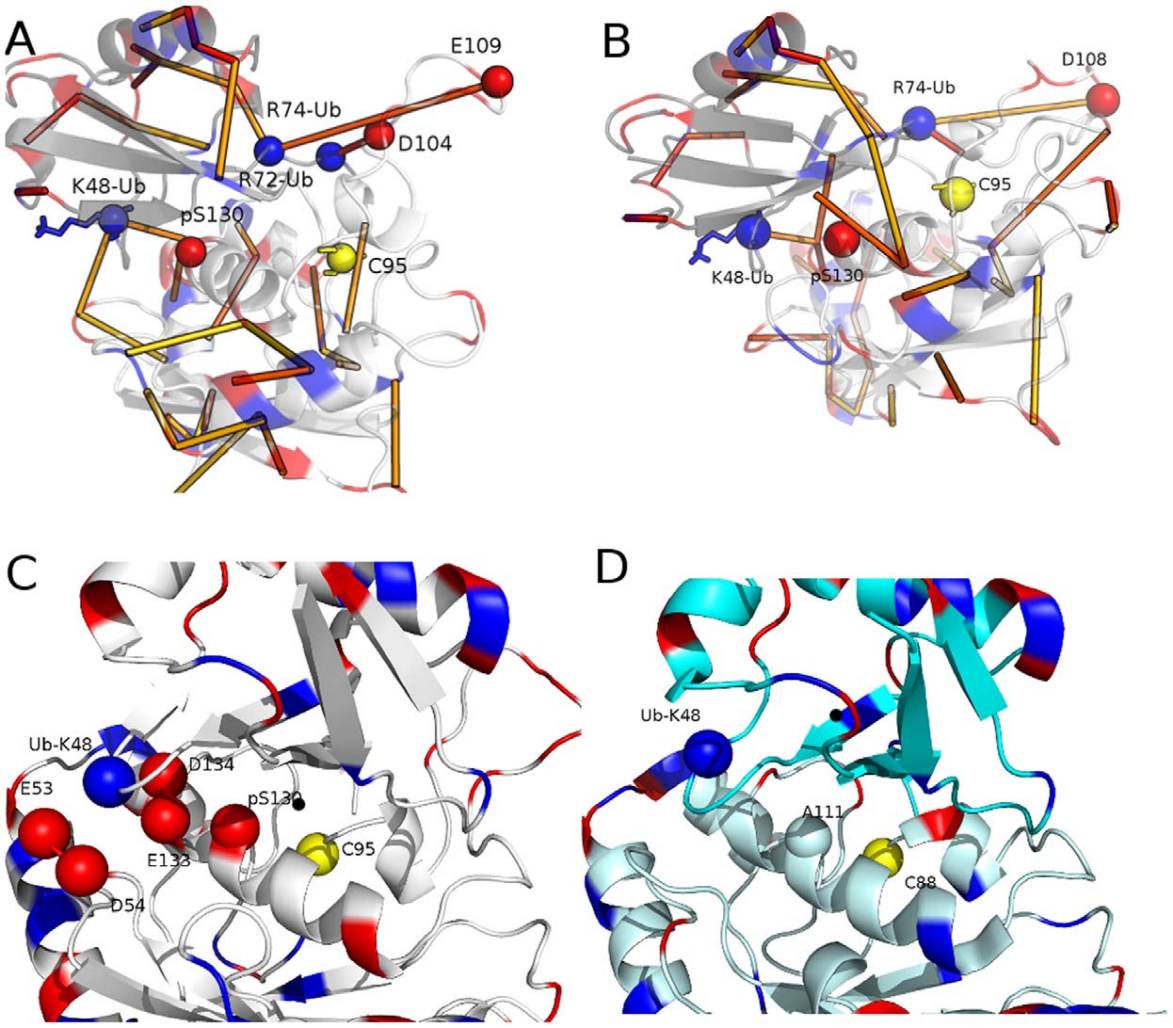
Ub-E2 family 3 complexes and the ternary complex between Ub-E2 and cognate E3 recognition domain. **A–B**) The salt bridge networks in the MD of E2-Ub complexes are reported as sticks coloured from yellow (low persistence) to red (high persistence). For sake of clarity, the salt bridges have been mapped on average structures of Cdc34-Ub complexes from the two different replicas. The catalytic cysteine, the positively charged Ub residues and the negatively charged L7 residues, as well as the phospho-S130 are indicated as yellow, blue and red spheres, respectively. **C–D**) the residues involved in the electrostatic intermolecular network between Ub K48 and Cdc34 in the Cdc34-Ub simulations (C), compared to the K48 surroundings in Ubc1-Ub simulations (D). The basic, acidic and the catalytic cysteine are shown as blue, red and yellow spheres, respectively. The A111 that replace the Cdc34 S130 in Ubc1 and E2 family 1enzymes is also indicated in the panel D.

To provide insights on the steps of the ubiquitination cascade upon E2 phosphorylation, MD simulations of Cdc34-Ub complexes were carried out and particular attention was devoted to the electrostatic intramolecular interaction networks. The L7 acidic residues are in a competent conformation for interaction with Ub, being in a suitable orientation for salt bridge interactions with the positively charged face of the Ub molecule (Figure 6A–B). In the E2-Ub complexes the hydrophobic residues of the L7 loop maintain the interactions with L4 and the L7 acidic residues rearrange to interact with the Ub molecule. In fact, interactions between L7 E109/D108 and D104 (Cdc34 numbering) with the C-terminal R72 and R74 of Ub can be detected with a persistence higher than 30% during the two Cdc34-Ub simulations (Figure 6A–B). In addition, the Cdc34 E113 can form a salt bridge interaction with Ub K11 with persistence around 20%. The interactions between E2 and in particular its acidic residue on this face of the Ub molecule, also concur also to long-range stabilize the side-chain orientation of the known Ub target lysine for poly-ubiquitination (Ub K48), which can form salt bridge interactions with other negatively charged residues of the E2 enzyme (mainly D54 (20.20%), E133 (51.28%) and D134 (61.60%)), as well as the phospho-S130 [39] (Figure 6A) in around 20% of the snapshots (Figure 6C). E133 and D134 belong to the acidic consensus for CK2 phosphorylation [54]. This cluster with a high density of close negatively charged residues in the E2-f3 (including also E53) can therefore form a network of electrostatic interactions with K48 in the E2-f3-Ub complexes (Figure 6C), which is likely to be a specific feature of E2-f3 enzymes or of E2 enzymes conserving the S130 phospho-site. In fact, in E2 enzymes not characterized by the acidic loop or conserved phospho-site at 130 position, as Ubc1-Ub [55], no stable electrostatic interactions involves K48 and the E2 enzyme, as judged by a 40 ns MD simulations of the complex (Figure 6D). Moreover, in Ubc1-Ub complex Ub has just two intermolecular salt bridge with the E2 enzyme (Ubc1 E117 and Ub R42 with 85.74% of persistence during the simulation) besides the one in the proximity of the thiolester bonds (E117-R72 with a persistence of 99.41%), in agreement with the fact that in solution the orientation of the Ub molecule within the E2 cavity was reported to be highly variable [34].

### The Acidic Loop also Provides an Interaction Interface for the Cognate E3

**Figure 7:**
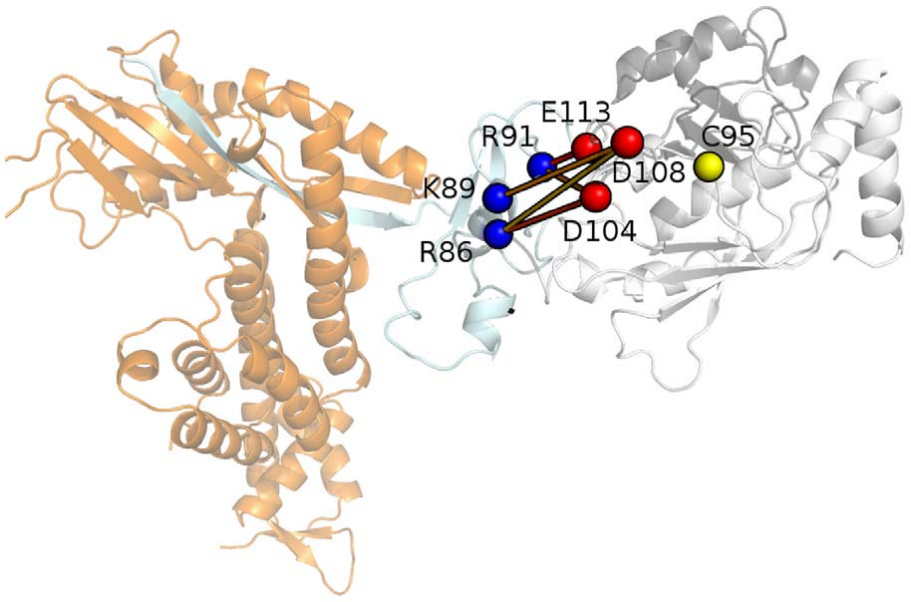
The Ub-Cdc34-E3 interface and the role of the acidic loop. The salt bridge networks between Rbx1 arginine and lysine residues (blue spheres) and the acidic L7 residues (red spheres) of Cdc34 is shown as turned out from a macro-trajectory of two independent 40 ns MD simulations. The Ub, Cdc34 and the Rbx1 domain are shown in black, light grey and light cyan, respectively. The rest of the E3 enzyme is indicated in orange. The salt bridge networks are reported as sticks coloured from yellow (low persistence) to red (high persistence).

Once the E2-Ub complex was formed, it has to be recruited by the cognate E3. We therefore provide details on the interactions involving L7 acidic loop in the ternary Ub-E2-E3 complex by molecular dynamics (MD) simulations. In particular, to assess the role of the L7 acidic loop in the recognition and interaction with the E3, a ternary complex between Cdc34-Ub and the domain Rbx1 (RING-box protein) of the cognate E3 RING was. obtained for homology model, using as template the 3D X-Ray structure of Clb (E3 domain)-Ubch7 (E2) complex (as described in the Method section). The RING domains of Rbx1 and Clb are structurally very similar, with a mainchain rmsd of 1.59 Å. Moreover, Ubch7-like and Cdc34-like E2s, even if belonging to two different E2 families according to the classification of Michelle et al. [31], share about 28% and 60% of sequence identity and similarity, respectively and when superimposed a mainchain rmsd lower than 1.70 Å, allowing to carry out a comparative modeling procedure for the ternary complex, as also recently applied to other complexes of E2-E3 and Ub [56]. L7 acidic residues can provide an interaction interface for the RING subdomain, thanks to electrostatic interactions with positively charged residues of Rbx1 (Figure 7). In particular, the L7 acidic residues D104, D108 and E113 (Cdc34 numbering) are in a conformation suitable to form salt bridge interactions with arginines and lysines of the Rbx1 domain (K89, R86, R91 and more rarely R99), creating an extended network of electrostatic interactions including all the partners of the ternary complex Ub-E2-E3 (Figure 7). These interactions are generally characterized by a high persistence (Table 2) in both the two independent MD simulations of the ternary complex, enforcing their relevance in mediating the interface between E2f3 catalytic domain and Rbx1. Interestingly, if the interactions exploited by loop 7 of UbcH7 and Clb RING domain are considered, they turned out to be mostly driven by hydrophobic interactions, as also recently pointed out by Chazin’s group for other UbcH7-E3 interactions [57]. In fact, they also demonstrated, by site-directed mutagenesis, that a SPA motif in loop 7 of UbcH7 and UbcH5 is required for a specific binding to the E3 ligase CHIP. The different nature of the intermolecular interactions exploited by L7 in Ubch7 and Cdc34 with their cognate RING domains enforces the notion that the acidic loop of Cdc34 can play an important role in the E2-E3 complexes. Several experimental data available in the literature can support this model of interaction between the acidic loop and Rbx. For example, in two different works it has been demonstrated that mutations in human Cdc34 of the Asp residues of the acidic loop affect the capability of assembly polyubiquitin chains on the target substrate [35], [36], an action that has to be mediated by interaction with the cognate E3. Moreover, in rabbit Ubc7, it was shown that a deletion of the acidic loop did not allow the E3-dependent conjugation to endogenous reticulocyte proteins [58].

**Table 2:** Salt bridge interactions between Rbx1 and the acidic loop in the MD Ub-Cdc34-E3 simulations.

	Replica 1(%)	Replica 2(%)	Macro-trajectory
ARG86_Rbx_-ASP104_Cdc34_	57.88	99.13	82.63
ARG86_Rbx_-ASP108_Cdc34_	58.05	/	23.22
ARG91_Rbx_ -ASP104_Cdc34_	90.7	36.5	58.19
ARG91_Rbx_-GLU113_Cdc34_	97.83	100	99.13
LYS89_Rbx_- ASP108_Cdc34_	/	67.16	47.30

## Discussion

The computational study here presented, integrating phylogenetic analyses to coarse-grained and atomistic MD simulations, shed a new light on the role of the acidic loop L7 in this group of enzymes.

At first, the 12–13 residue insertion in L7 turns out to be an ancestral and conserved motif, characterized by an invariant alternance of hydrophobic and acidic residues in all the family 3 members, independently on the sub-family (Figure 1B). In fact, here we demonstrated that the L7 acidic loop of Cdc34-like enzymes is a conserved functionally relevant omega loop in family 3 E2s. At first, our results enforce the notion that L7 provides a general regulatory mechanism, acting as a “lid” which modulates the accessibility of the catalytic site and impairs Ub-charging capability of E2-f3 enzymes, at least until a conformational change is promoted by an external signal, as post-translational phosphorylation [39] or the interaction with the cognate partners in the cascade [19].

The analyses here discussed and their interpretation in the context of the available literature [35]–[37], more importantly, point out a functionally relevant role of L7 of E2-f3 not only in Ub-charging steps, but in all the different steps of the ubiquitination cascade. In fact, the strict conservation and spatial alternation of hydrophobic and acidic residues in L7 has a functional role, providing the enzymes with the possibility to regulate several different intra- or intermolecular interactions required in the different steps of the ubiquitination cascade. The alternance of hydrophobic/acidic residues may be an ancestral characteristic of E2 enzymes that has been lost during evolution or gene duplication in several E2 families specifically involved in different functions with respect to the Cdc34-like enzymes. This is attested by bacterial E2-like enzymes featuring a Cdc34-like acidic insertion, as well as by the existence of reminiscences of the acidic insertions in other E2 families, as E2s involved in sumoylation (family 7) or UbcH7 itself (family 4), here employed as template.

**Figure 8:**
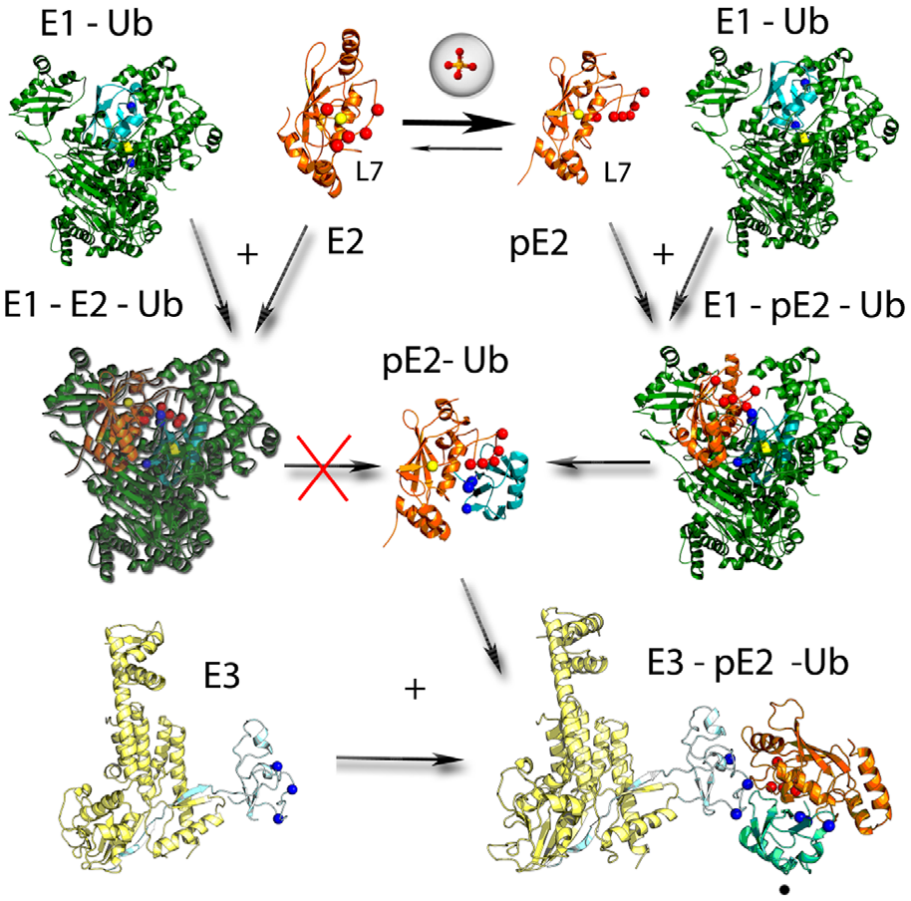
A model of the role of L7 in the different steps of the ubiquitination pathway in Cdc34-like enzymes. E1, E2 of family 3, and the ubiquitin are shown in green, orange and cyan, respectively. The structure of the E3 component which includes the Rbx1 domain is shown in light cyan and the rest of E3 in light orange. The acidic residues in loop L7 of family 3, as well as the phospho-S130 of E2 are indicated as red spheres. The basic residues of Ub C-terminal tail and on the Rbx1 interaction interface for E2 are shown as blue spheres. E1 enzyme is shown in its experimentally determined covalently bound to Ub in the upper right and left region of the panel (PDB code: 3CMM, Ub from the complex Ubc1-Ub is shown), the other structures have been derived by our investigation. E2 enzyme of family 3 exists in both closed inactive and open active conformations of L7, with closed conformation stabilized by hydrophobic interactions between L7–L8. Phosphorylation of E2 enzyme stabilized the active conformation of L7 and makes it competent for ubiquitin transfer, so that the ubiquitin can bind to E2 and this determines the progression of the ubiquitination cascade (right side). The effect is induced by electrostatic repulsion between phospho-S130 and the L7 acidic residues and in open conformation hydrophobic L7 residue stabilize the interface for E3 binding on L4. The lack of an open L7 conformation does not permit the transfer of Ub from E1 cavity to the E2 catalytic cysteine. Once the E2-Ub complex is formed (centre), acidic L7 residues can rearrange to interact by salt bridges with C-terminal tail of Ub. The last of the ubiquitination cascade require the recruitment of E3 enzyme, where L7 can provide by its acidic residues an additional and specific interaction interface for arginine residues of Rbx1.

A general model can be described for L7 involvement in the different stages of the ubiquitination pathway of family 3 E2s (Figure 8), integrating our data to the experimental ones available from the literature [19], [35]–[39], [59]. In fact, in absence of a phosphorylation event [39] or the interaction with the cognate partners [19], Cdc34-like enzymes feature a structural ensemble in which both partially open and closed conformations of L7 co-exist, with the latter being the prevailing native state. L7 hydrophobic residues can provide the necessary stabilization of the closed states by hydrophobic interactions with residues surrounding the ubiquitin binding cleft and they can shield the catalytic cysteine from not specific interactions with ubiquitin (Figure 8). In fact, we also previously showed that L7 activation is an event that has to precede the interaction with E1 Ub-activating enzyme, since until L7 is displaced toward an open conformation, the E2 ubiquitin charging cannot take place [39] (Figure 8). Therefore, in our model, the interaction of inactive Cdc34-like E2s with the cognate E1, which has already recruited the Ub molecules, will not provide E2-Ub complexes and the continuation of the Ub cascade.

The mobility of the acidic loop and its modulation of the solvent accessibility of Cdc34 catalytic cleft can also explain the low basal ubiquitin-charging by Cdc34 in absence of the CK2 kinase which has been observed *in vitro* [38]. CK2-mediated phosphorylation events can ensure the correct timing for Cdc34-like E2s activation, affecting not directly the acidic loop but distal sites, as S130 of the UBC domain (which is always fully-solvent exposed for phosphorylation, according to our previous studies [39]), and long-range transmitting the effects to the acidic loop. Also multiple phosphorylation events in the C-terminal accessory domain of some Cdc34-like enzymes are known and they still require further investigations at the structural level, but are more likely involved in the regulation of the last steps of ubiquitination which require E3 recruitment [60]. In fact, post-translational phosphorylation is a ubiquitous mechanism for cellular regulation and it is known to modulate protein conformations by changing their energy landscape. These effects are largely driven by the electrostatic perturbation induced by the phosphate group [61], as demonstrated by other examples of phosphorylations which long-range transmit an effect to protein active sites [62]–[65]. Regulation by phosphorylation of Cdc34-like enzymes well fit with this scenario. We recently showed that not only phosphorylation at the catalytic domain of E2-f3 can stabilize the whole protein structure, but more importantly it can activate the Cdc34-like enzymes transmitting its effects to the L7 acidic loop [39]. In this phase, the L7 acidic residues are involved that can promote open conformations of L7, by repulsive electrostatic effects induced by the phospho-serine pS130 and therefore provide the access of Ub to the active site and a competent E2 conformation for the ubiquitin charging (Figure 8). Only open conformations of L7 are suitable for Ub charging as highlighted by both cavities detection during dynamics of the native proteins and the MD simulations of Cdc34-Ub complexes. In this competent form for the Ub-charging, L7 of Cdc34-like enzymes populates conformational states in which its hydrophobic residues stabilize the open conformations by hydrophobic interactions with L4, whereas the L7 acidic residues are solvent-exposed. Upon Ub-charging the acidic residues can rearrange in order to further stabilize the conformation of Ub within the catalytic cleft by electrostatic interactions with the C-terminal Ub arginines (Figure 8). This is a remarkable difference with respect to other E2 enzymes lacking the acidic insertion, for which heterogeneous and highly flexible binding modes have been shown in solution [30] and for which the activity or specificity can be related to the function of other E2 domains, as in the case of Ubc1 [66], or even to the interactions with other regulatory proteins or even Ubl modification of the E2s [30], [67]. The presence of the intermolecular salt bridges in Cdc34-Ub complexes, which act as an electrostatic “Velcro”, is likely to allow, on the opposite Ub side, a stabilization of Ub K48 within an intermolecular electrostatic network with negatively charged residues well-conserved in E2-f3 enzymes. Interestingly, it has to mention that in other E2 enzymes, lacking the acidic L7 insertion, a higher flexibility of the Ub molecules in the E2 pocket has been pointed out [34]. A lack of stable intermolecular interactions between Ub and the E2 cavity once the Ub molecules is bound, is also strengthened by our MD simulations of the Ubc1-Ub complex, which lacks the acidic insertion in L7. Phospho-S130, which is a coevolving site with the acidic loop [39] and less conserved in other E2 families, is located at the interface with K48 and could guarantee a further stabilization to the K48 orientation. L7 seems therefore not only a lid structure that has to be activated to begin the ubiquitin-charging activity. It has a more complex and important role in driving other intermolecular interactions in the other steps of the ubiquitination cascade, and this rely on the conservation of not only negatively charged residues but of a group of hydrophobic residues, both of them exploiting different function in the different steps.

This scenario also allows to clarify experimental evidences on mutants of the L7 acidic residues in both yeast and human Cdc34 enzymes, as well as in Ube2g2 [35], [36], [68]. In fact, the central role of acidic residues in reducing the conformational freedom of the E2-bound Ub and in acting as an interface for salt bridge networks with both E3 and Ub, suggested by our simulations, explain the fact that mutations of these acidic residues abolished the correct polyubiquitin chain assembly [68], as well as affect the processivity and the chain synthesis with a correct K48-polyubiquitin topology [35], [36]. They are all steps related to E2-E3 interactions and in this context our data can complement the present available scenario and provide a rational, at the molecular level, of the deleterious effects induced by mutations of the acidic residues of L7 in Cdc34 on processivity of the reaction, as well as capability to form K48-linked polyubiquitin chains and the proper Ub transfer [35], [36], [59]. In particular the effects experimentally observed when several of the conserved L7 acidic residues are mutated to alanine, well fit with the simulation data here provided, where these residues has a central role in the intermolecular electrostatic network present in both the binary and ternary complexes. In fact, mutation of these acidic residues is likely to affect the proper intermolecular interface between E2-Ub and the E3 enzyme and to cause a less specific and stable interaction interface for E3 recruitment, which in turn can translate in the detrimental effects induced by L7 mutations or deletions on the polyubiquitination and Ub transfer.

In fact, to complete the model here formulated, our results also points the involvement of the L7 acidic residues in providing an interface for interactions with specific domains of the cognate E3 (Figure 7 and Figure 8). In this context, L4 residues are known to generally mediate interactions with both RING or HECT E3s, in the E2 superfamily [31]. In the case of Cdc34-like enzymes, hydrophobic L7 residues stabilize the L4 conformation for E3 interactions, decreasing L4 conformational freedom by the hydrophobic cluster, and on the other side the conserved acidic L7 residues confer specificity for interactions with RING subdomains bearing positively charged residues. In fact, Rbx1 is rich of arginine residues at the interface with Cdc34 and the L7 acidic residues can act as linker between these charged residues and Ub arginines creating an extend intermolecular electrostatic network.

In summary, our investigation shows the molecular details underlying the pivotal role of L7, not only in influencing ubiquitin transfer from E1 to E2 and ubiquitin-charging on the E2 catalytic cysteine, but also in the downstream events related to ubiquitin chain assembly, which require the interaction with E3 enzymes.

## Materials and Methods

### Sequence Alignments and Phylogenetic Analysis

Starting from a preliminary set of sequences of E2 UBC domains of family 3 [31], additional family 3 sequences were searched by DivergentSet [69], screening the NCBI non-redundant protein database. PSI-Blast [70] was employed with E-value cut-off of 1e^−20^ and a H-value cut-off of 1e^−20^, in order to find E2 sequences isolated from different organisms, spanning as much as it possible the tree of life. Redundant sequences and non-informative pseudogenes were discarded. The sequences set was further pursued discarding sequences from prokaryotic organisms, according to ref. [31].

Multiple sequence alignment of UBC domain of family 3 E2s were performed by ClustalW [71] and T-Coffee [72], comparing about 100 E2 sequences. In particular, the sequences corresponding to the UBC domain of each E2s have been determined by intra-family sequence alignments with family 3 members for which known 3D experimental structure are available. The residue conservation in the acidic loop and its immediate surroundings (residues 92–110 in human Ube2g2) was evaluated with different scoring functions and average conservation values for each residue was calculated [73]. In particular, entropic scores using Shannon’s information theoretical entropy, as well as matrix-based and sequence weighted scores have been employed. Calculation of conservation degree were carried out with each of these methods using both Blosum62, Blosum45 and modified PET91 scoring matrices. Moreover, a consensus pattern for the acidic loop region was calculated, both with a 70% and 80% threshold of conserved residues in the aligned sequences.

For phylogenetic analysis, bootstrapping of 100 replicates was performed on the multiple alignment according to the Felsenstein method, whose parameters were set on default, using the PHYLIP Package version 3.69. The set of replicates was used to generate distance matrices based on Jones-Taylor-Thornton matrix model, which have been successively used to generate unrooted phylogenetic trees according to the Fitch-Margoliash distance matrix method. A consensus tree was finally generated and visualized by TreeIllustrator version 0.52 Beta.

### Homology Modelling

The known experimental 3D structures of members of E2-f3 were retrieved from the Protein Data Bank (PDB) using BLAST-p similarity searches. 6 structures turns out (PDB entries: 1PZV, 3FSH, 2UCZ, 2OB4, 2CYX and 2KLY); 5 solved by X-ray crystallography and a recently deposited NMR structure. They refer to 5 different E2s: *C.elegans* Ubc7 (CeUbc7), *Mus musculus* Ube2g2 (MmUbe2g2), *Saccharomyces cerevisiae* Ubc7 (Ubc7), human Cdc34 (hCdc34), human Ube2g2 (Ube2g2).

In order to increase the subset of protein structures to analyzed, 4 homology models have been generated for yeast Cdc34 (Ubc3), *Saccharomyces pombe* Ubc3 (SpUbc3), *Arabidopsis thaliana* Ubc7 (AtUbc7), and *Drosophila melanogaster* Dm_CG40045 E2, using as templates the known X-ray structures of Ube2g2 [PDB entry 2CYX], Ubc7 [PDB entry 2UCZ] and CeUbc7 [PDB entry 1 PZV], which generally share with the targets 40–60% of sequence identity. Multiple sequence alignments between target sequences and the templates with known 3D structure were performed with ClustalW and T-Coffee. The multiple alignments were compared and modified by hand according to information on functional residues and secondary structures to get the optimal alignment for homology modeling, which has been carried out by Modeller v. 9.9.

Homology models of the complexes of Cdc34-Ub and Ube2g2-Ub have been created using as template structures Ubc1-Ub (pdb entry, 1FXT) and further details are provided in the Text S1.

The ternary complex between Cdc34-Ub and Rbx1 has been obtained by homology using as template the orientation of the complex between UbcH7 and its cognate E3 RING (c-Clb) (pdb entry 1FBV), as well as the X-ray structure of Rbx1 RING sub-domain from pdb entry 3DQV. In particular, structural alignment of Cdc34 and UbcH7, as well as of Rbx1 and c-Clb RING sub-domains were perfomed with Dali and the alignments were used to generate a ternary model by Modeller v.9.9. Two independent 40 ns MD simulations were then carried out on the ternary complex to better describe the electrostatic interactions at the Ub-Cdc34 and Rbx1 interface, as well as their persistence in the MD ensemble.

### Molecular Dynamics Simulations

Molecular dynamics (MD) simulations were performed using GROMACS 3.3 and 4 (www.gromacs.org), implemented on a parallel architecture with the GROMOS96 force field and also validated for some sample proteins using the recently released AMBER ff99SB-ILDN force field [74].

The three Cdc34 models [39], the X-ray structures of Ubc7 and Ube2g2, as well as NMR structure of Ube2g2, were used as starting point for all-atom MD simulations. Additional simulations were carried out for the Cdc34-Ub, Ubc1-Ub and Cdc34-Ub-E3 complexes (Table 1). The starting structures were soaked in a dodecahedral box of SPC (Gromos96 force field) or TIP3P (Amber ff99SB-ILDN force field) water molecules and simulations were carried out using periodic boundary conditions. Productive MD simulations were performed, upon several solvent equilibration, termalization and pressurization steps, in the isothermal-isobaric (NPT) ensemble at 300 K and 1 bar with a 2 fs time-step. Electrostatic interactions were calculated using the Particle-mesh Ewald (PME) summation scheme. Van der Waals and Coulomb interactions were truncated at 1.0 nm. The non-bonded pair list was updated every 10 steps and conformations were stored every 4 ps.

To improve the conformational sampling, several independent simulations were carried out for each protein or complex, initializing the MD runs with different initial atomic velocities. In the following, MD trajectories collected for the same system at the same temperature, but characterized by different initial atomic velocities, are referred to as replicas (Table 1). The replicas collected for each system differ for duration and number, spanning from 2 (in the case of Ub-E2-E3 complexes) to 18 (in the case of native Cdc34 simulations), and are summarized in Table1, allowing to collect more than 3 µs of simulations.

### Analysis of MD Simulations

The main chain root mean square deviation (rmsd) was computed using the starting structures as a reference and its time evolution was monitored, along with the evolution of protein radius of gyration. From 2 to 10 ns of each replica were required to reach stable Rg and main chain rmsd values and, therefore conformations collected in the first part of simulations according to the convergence of rmsd profiles, were discarded to ensure that calculated parameters reflect the intrinsic properties of each system. For each system, the equilibrated portions of each replica were joined together in macro-trajectories, which are representative of different directions of sampling around the starting structures. The secondary structures were calculated by the DSSP program, whereas the solvent accessibility by Naccess and the GROMACS g_sas tool. The Cα root mean square fluctuation (rmsf) per residue from the average structure was calculated on trajectories filtered on the principal components which describe more than 70% of the essential space (see below). In order to verify the consistency of rmsf profiles, the Pearson correlation coefficient was calculated comparing rmsf data sets relative to the replicas of the same system. The correlation coefficients are generally higher than 0.65, indicating that the collected simulations give a consistent picture of protein flexibility.

CAST-p (Computed Atlas of Surface Topography of proteins) [48] was used to identify pockets and cavities in snapshots from the MD simulations using a solvent probe of 1.4 Å radius.

### Principal Component Analysis (PCA)

PCA reveals high-amplitude concerted motion in MD trajectories, through the eigenvectors of the mass-weighted covariance matrix (C) of the atomic positional fluctuations [75], which was calculated on protein Cα of the single replicas and the macro-trajectories. In our macro-trajectories, the first three eigenvectors describe more than 60% of the total motion. The cosine content and the root mean square inner product [76] on the single replicas and randomly concatenated macro-trajectories have been calculated to validate the achieved conformational sampling, according to a procedure previously developed [77].

### Dynamical Cross-correlation Matrices (DCCM) in MD Simulations

Correlation plots were obtained by computing Cα dynamical cross-correlation matrices (DCCM) *C(i,j)* [78], using non over-lapping averaging windows of 1 ns, and also compared, for validation, to correlations on averaging windows of 4 and 10 ns. Only the most significant (|*C(i,j)*|>0.35) long range (|*i−j*|>10) positive and negative correlations have been considered. The sequence cutoff has been selected to exclude correlations relative to the inner α-helix structures and contiguous in the primary sequence. Moreover, since we discuss average *C(i,j)* matrices, a cutoff of 0.35 (in absolute value) for significant correlations has been selected to exclude pairs of residues which are poorly communicating and likely to be characterized by uncoupled motions. The cut-off has been selected evaluating the distribution of the correlation values in the MD ensemble. Moreover, to carefully verify that the analysis of an average *C(i,j)* matrix does not cause a loss of relevant information, the consistency between the average *C(i,j)* matrix with the individual matrices used in the averaging has been evaluated. Correlations were then plotted on the 3D structures by connecting atoms *i* and *j* with lines, with thickness proportional to *C(i,j)*.

### Electrostatic and Hydrophobic Interaction Networks in the MD Ensemble

The salt bridge interactions have been evaluated as oppositely charged groups at less than 0.45 nm of distance in at least 20% of the macro-trajectory frames. For hydrophobic interactions instead a distance cutoff of 0.55 nm was used. The angles between the groups involved in the electrostatic interactions have been also checked. The persistence cut-off of 20% was selected as the persistence value which best divided the interaction dataset in well-separated groups, defines as signal and noise, according to a protocol previously applied [79]. To well identify on the 3D structure networks of salt bridges and hydrophobic interactions, the residues involved in the interactions have been represented as nodes of an unrooted unoriented graph, in which two nodes were connected by arcs if a salt bridge was identified between them.

### Normal Mode Analysis (NMA), Brownian Dynamics (BD) and Discrete Molecular Dynamics (DMD) Sampling

The analyses of chained cross-correlations and B-factors have been carried out for the 6 experimental structures and the 4 homology models of E2-f3 enzymes. In particular 3 different coarse-grained algorithms have been used: normal mode analysis (NMA), Brownian dynamics (BD) and discrete dynamics (DMD), as they are implemented in FlexServ [41], which assumes a coarse-grained representation of the protein (Cα-only). In particular, NMA and BD consider that the inter-residue interactions are controlled by a harmonic-like potential energy expressed as:
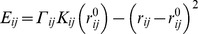
Where *r_ij_* is the distance between residues *i* and *j,* and *r^0^_ij_* the equilibrium value, *Г_ij_* the Kirchhoff connectivity matrix, and *K_ij_(r^0^_ij_)* the stiffness force constant. In a pure harmonic model *K_ij_(r^0^_ij_)* has a single value *K_ij_* and *Г_ij_* = −1for atoms within a given distance cutoff and 0 otherwise. Flexserv implements NMA within the Anisotropic Network Model (ANM) formalism, which through the diagonalization of the hessian matrix provides eigenvalues and eigenvectors that not only describe the frequencies and the shapes of the normal modes, but also their direction [80]. The Kovacs, inverse exponential formalisms, in which the force costant is defined by a continuous function have been employed, in which K_ij_(r^0^_ij_) = C (r*/r^0^_ij_)^6^ where C is the stiffness costant (C = 40 kcal/molÅ^2^) and r* is a fitted constant, taken as the mean Cα-Cα distance between consecutive residues (r* = 3.8 Å) [41]. In BD, the potential energy used to compute forces assumes a quasi-harmonic representation of the interactions similar to that suggested by Kovacs et al. (ref)



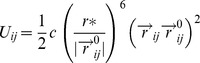



The initial condition is the native structure that is supposed to be in the minimal energy state, from which the relative vectors *r_ij_^0^* are computed. Factor C and r* are equal to that discussed above for NMA. The mass of all Cα atoms is set to 100 dalton (i.e. that of an average residue). The velocity-dependent friction of water (0.4 ps^−1^) is considered. 1 fs time step and 5000000 steps have been used for BD calculations.

DMD assumes that residue-residue interactions are controlled by infinite square wells centered at equilibrium distance with width fitted to reproduce flexibility in atomistic details. In the context of this approach, a particle is either moving at a constant velocity or colliding with a wall, allowing the derivation of trajectories using ballistic equations. The interaction potentials are defined as infinite square wells so that the particle-particle distances vary between *(1−σ)r_ij_^0^* and *(1+σ)r_ij_^0^* with *r_ij_^0^* being the distance in the native conformation and 2σ the width of the square well. Residue-residue interaction potentials are defined only for the particles at a distance smaller than a cut-off radius *r_c_* in the native conformation, which is set to 8 Å. σ is set to 0.1, while for consecutive pairs of residues a smaller well width (σ = 0.05) was selected to keep the Cα-Cα distances closer to the expected value 3.8 Å.

Chained correlations among residues have been calculated post-processing the raw dynamical correlation matrix. Hydrophobic residues in the L7 loop (residues 92–110, Ube2g2 numbering) have been selected as root residues with a correlation threshold of 0.5 and the iterative search for chained correlation has been carried out till a depth of 5. B-factors which determined oscillations of residues with respect to their equilibrium positions were calculated according to FlexServ procedure [41]. Hinge point prediction has been carried out to identify putative hinge points in the acidic loop, using the B-factor slope change method as implemented in FlexServ [41].

## Supporting Information

Figure S1 **Multiple sequence alignment of family 3 E2 representative members used for the phylogenetic investigation.** Identical residues (red-filled boxes) and similar residues (red boxes) are indicated.
(PDF)

Table S1 **Sequences of E2 enzymes belonging to family 3 and their division in sub-families R, R1, R2, G1, G2 and #, along with the corresponding entries in the UNIPROT and NCBI databases.**
(XLS)

Table S2 **Consensus of chained correlations derived by NMA, BD and DMD methods, as implemented in FlexServ, using as root residues the hydrophobic residues of L7.**
(PDF)

Text S1 **This file contains the following supporting figures for this article:** **Figure 2** **.** B-factor profiles obtained for the E2 representative members of family 3 by NMA, BD and DMD. Figure 3. Rmsf profiles from MD simulations. The 3D structure of each E2 family 3 enzyme is used as a reference and each residue colored with different shade of colors according to Cα rmsf values calculated from the MD simulations (from light to dark colors for increasing rmsf values). **Text 1. Modelling of the E2-Ub complexes.** **Figure 4** **.** Ubiquitin position and orientation is shown to be different in the three known complexes with E2 enzymes (PDB code 1FXT, 3A33, 2KJH). **Figure 5** **.** (A) E1 catalytic subunit SAE2 and Ubc9 have been crystallized in a structure with PDB code 2PX9 and it is the E1 structurally more similar to the Uba1 E1 enzymes for Ub. (B/C/D) Different ubiquitin positions have been obtained from the three known E2-Ub complexes through structural superimpositions. The only orientation that satisfies the spatial restraints imposed by the E1 structure is the one that appears in 1FXT E2-Ub complex (B). **Figure 6** **.** (A) UBA3 (E1) and Ubc12 have been crystallized in a structure with PDB code 2NVU. (B/C/D) Different ubiquitin positions have been obtained from the three known E2-Ub complexes through structural superimpositions. The only orientation that satisfies the spatial restraints imposed by the E1 structure is the one that appears in 1FXT E2-Ub complex (B). **Figure 7** **.** A preliminary model of an E1–E2 (family 3)-Ub complex has been obtained by superposing UBA1 structure (PDB code: 3CMM) to SAE2 structure (PDB code: 2PX9), using ubiquitin position as it appears in the complex Ubc1-Ub (1FXT). The catalytic cysteine of the E2 enzyme is indicated as yellow sphere and the E1 catalytic cysteine as yellow stick. Acidic L7 and phospho-S130 residues are shown as red spheres and the positively charged residues of Ub C-terminal chain as blue spheres.
(PDF)
