# Supplementary material for: Loop 7 of E2 Enzymes: An Ancestral Conserved Functional Motif Involved in the E2-Mediated Steps of the Ubiquitination Cascade
Source: PLoS One. 2012 Jul 18;7(7):e40786. doi: 10.1371/journal.pone.0040786 (PMC3399832; doi:10.1371/journal.pone.0040786)
Supplement: Figure S1 — Multiple sequence alignment of family 3 E2 representative members used for the phylogenetic investigation. Identical residues (red-filled boxes) and similar residues (red boxes) are indicated. (PDF) [file pone.0040786.s001.pdf]

```
                                  α1                      β1               β2              β3
Hs_Ube2G2                 QQQQQQQQQQQQQQQ         TT  ------>   TT  TT---------->   TT        ------->

Hs_Ube2G2      1  .......MAGTALKRLMAEYKQLTLN.PPEG.IVAGPMNEENFFEWEALIMGP.EDTCFEFG.VFPAILS
Mm_Ube2G2      1  .......MAGTALKRLMAEYKQLTLN.PPEG.IVAGPMNEENFFEWEALIMGP.EDTCFEFG.VFPAILS
Rn_G2          1  .......MAGTALKRLMAEYKQLTLN.PPEG.IVAGPMNEENFFEWEALIMGP.EDTCFEFG.VFPAILS
Md_G2          1  .......MAGTALKRLMAEYKQLTLN.PPEG.IVAGPMNEENFFEWEALIMGP.EDTCFEFG.VFPAILS
Gg_G2          1  .......MAGTALKRLMAEYKQLTLN.PPEG.IVAGPMNEENFFEWEALIMGP.EDTCFEYG.VFPAILS
Xl_G2          1  .......MAGTALKRLMAEYKQLTLN.PPEG.IVAGPINEENFFEWEALIMGP.EDTCFECG.VFPAILS
Xt_G2          1  .......MAGTALKRLMAEYKQLTLN.PPEG.IVAGPINEENFFEWEALIMGP.EDTCFECG.VFPAILS
Dr_G2          1  .......MAGTALKRLMAEYKQLTLN.PPEG.IVAGPVNEENFFEWEALIMGP.EDTCFEGG.VFPAILS
Tn_G2          1  .......MAGTALKRLMAEYKQLTLN.PPEG.ILAGPPNEENFFEWEALIMGP.QDTCFEGG.VFPAVLS
Nv_G2          1  .......MAGFALRRLMAEYKQLTVN.PPEG.IMAGPVNEENFFEWEALITGP.EGTCFEGG.IFPAKLV
Dm_G2          1  .......MAGSALRRLMAEYKQLTLD.PPEG.IVAGPISEDNFFEWEALIAGP.EGTCFEGG.VFPARLI
Dp_G2          1  .......MAGSALRRLMAEYKQLTLD.PPEG.IVAGPVSEDNFFEWEALIAGP.EGTCFEGG.VFPARLV
Aa_G2          1  .......MAGSALRRLMAEYRQLTLN.PPEG.IIAGPVSEENFFEWEALITGP.EGTCFEGG.VFTAKLV
Tc_G2          1  .......MAGSALRRLMAEYKQLTLN.PPEG.IIAGPINEENFFEWEALITGP.EGTCFEGG.VFPAKLI
Ce_Ubc14       1  .......MAGYALKRLMTEYKELTTR.PPEG.IIAAPIDEDNFFEWECLITGP.EETCFANG.VFPARIT
Cb_G2          1  .......MAGYALKRLMTEYKELTTR.PPEG.IIAAPIDEDNFFEWECLITGP.EETCFANG.VFPARIT
Cp_G2          1  .......MAGSALRRLMAEYRQLTLN.PIDS.IIAGPISEENFFEWEALITGP.EGTCFEGG.VFTAKLV
Am_G2          1  .......MAGSALRRLMAEYKQLTLN.PPEG.IIAGPINEENFFEWEALITGP.EGTCFEGG.VFPAKLI
Sp_Ubc3        1  ......MSKAMALRRLMKEYKELTEN.GPDG.ITAGPSNEDDFFTWDCLIQGP.DGTPFEGG.LYPATLK
Sc_Ubc7        1  .......MSKTAQKRLLKELQQLIKD.SPPG.IVAGPKSENNIFIWDCLIQGP.PDTPYADG.VFNAKLE
Cg_G2          1  .......MSKTAQKRLYKELQQLIKD.SPPG.IVAGPKSDDNLFVWDCLIQGP.PDTPYAGG.VFNAQLE
Vp_G2          1  .......MSKTAQKRLFKEYQQLIRD.SPPG.IVAGPANENDLFLWDCLIQGP.PDTPYEHG.VFNAQLR
Asg_G2         1  .......MSKTAQKRLMKELQQLLRD.SPDG.IVAGPVSEDNLFLWDCLIEGP.ADSPYEGG.VFNARLQ
Pg_G2          1  ......MPRTTAQRRLLKEFQQLSRD.PPEG.IIAGPVSENDLYKWECLLEGP.TDTPYENG.VFPAILE
Dh_G2          1  .....MAPRSTAQKRLLKEYQQLARD.PPPG.IVAGPISEDDLFKWECLLAGP.PDTPYENG.VFPASLT
Ps_G2          1  .....MAPRSTAQKRLLKEYQQLARD.APPG.IVAGPVSEDDLFKWECLLEGP.PDTPYENG.VFPATLS
Ca_G2          1  .....MPPRSTAQKRLLKEYQQLSRD.PPPG.IIAGPVSEDNLYKWECLLEGP.SDTPYENG.VFPAVLT
Le_G2          1  .....MPPRSTAQKRLLKEYQLLTRD.PPPG.IIAGPVLEDNLFKWECFLEGP.VDTPYENG.VFPAILT
Hs_Ube2G1      1  ......MTELQSALLLRRQLAELNKN.PVEG.FSAGLIDDNDLYRWEVLIIGP.PDTLYEGG.VFKAHLT
Mm_Ube2G1      1  ......MTELQSALLLRRQLAELNKN.PVEG.FSAGLIDDNDLYRWEVLIIGP.PDTLYEGG.VFKAHLT
Rn_Ube2G1      1  ......MTELQSALLLRRQLAELNKN.PVEG.FSAGLIDDNDLYRWEVLIIGP.PDTLYEGG.VFKAHLT
Md_G1          1  ......MTELQSALLLRRQLTELNKN.PVEG.FSAGLIDGNDLYRWEVLIIGP.PDTLYEGG.VFKAHLT
Gg_G1          1  ......MTELQSALLLRRQLAELNKN.PVEG.FSAGLIDDNDLYRWEVLIIGP.PDTLYEGG.VFKAHLT
Xl_G1          1  ......MTELQSALLLRRQLAELNKN.PVEG.FSAGLIDDNDLYRWEVLIIGP.PDTLYEGG.VFKAHLT
Xt_G1          1  ......MTELQSALLLRRQLAELNKN.PVEG.FSAGLIDDNDLYRWEVLIIGP.PDTLYEGG.VFKAHLT
Dr_1_G1        1  ......MTEPQSALLLRRQLAELNKN.PVEG.FSAGLIDDNDLYRWEVLIIGP.PDTLYEGG.VFKAHLT
Dr_2_G1        1  .......MTEQSALLLRKQLAELNKN.PVEG.FSAGLIDDDDIYQWEVVVIGP.QDTMFEGG.FFKAHLI
Tn_1_G1        1  .......MTAPSALLLRKQLAELNKN.PVEG.FSAGLVDDDNIYRWEVVVMGP.QDTLFEGG.FFRAILT
Tn_2_G1        1  ......MTEPRSALLLRRQLTELNKS.PVDG.FSAGLIKDNDLYRWEVMIIGP.PDTLYEGGGVFKAHLT
Nv_G1          1  ......MSEPQSALLLRKQLAELNKN.PVEG.FSAGLIDDNDIYRWEVLIIGP.PDTLYEGG.FFKAHLQ
Dm_CG40045     1  ......MSELQSSLLLKKQLAELNKN.PVEG.FSAGLIDENDIFRWEVLIIGP.PDTLYEGG.FFKAHLY
Dm_CG9602      1  ......MSELQASLLLNRQLSELQRH.PVEG.FSAGLVSDSDIFKWEVVIIGP.PDTLYEGG.FFKAHLI
Dp_G1          1  ......MSEQQASLLLRRQLTELHRN.PVEG.FSAGLINDADIFKWEVVIIGP.PETLYEGG.CFKAHLI
Aa_G1          1  ......MSEPQSSLLLKKQLAELSKN.PVEG.FSAGLIDDNDIFRWEVLIIGP.PDTLYEGG.FFKAHLH
Tc_G1          1  ......MSEPQSALLLRKQLAELNKN.PVEG.FSAGLIDDNDIYRWEVLIIGP.PDTLYEGG.FFKCHLY
Ce_Ubc7        1  ........MEQSSLLLKKQLADMRRV.PVDG.FSAGLVDDNDIYKWEVLVIGP.PDTLYEGG.FFKAILD
Cb_G1          1  ........MEQSSLLLKKQLADMRRV.PVDG.FSAGLVDDNDIYKWEVLVIGP.PDTLYEGG.FFKAILD
Sp_Ubc15       1  ......MPSSASEQLLRKQLKEIQKN.PPQG.FSVGLVDDKSIFEWEVMIIGP.EDTLYEGG.FFHATLS
Os_1_#         1  ....MATAASQASLLLQKQLKDLAKN.PVDG.FSAGLVDDSNVFEWQVTIIGP.PDTLYDGG.YFNAIMT
Os_2_#         1  ....MAATISQASLLLQKQLKDLAKH.PVDG.FSAGLVDDSNVFEWQVTIIGP.PDTLYDGG.YFNAIMS
Zm_1_#         1  ....MATTTTQASLLLQKQLRDLAKH.PVDG.FSAGLVDDSNVFEWQVTIIGP.PDTLYDGG.YFNAIMS
Zm_2_#         1  ....MASSASQASLLLQKQLKDLAKN.PVDG.FSAGLVDDSNIFEWQVTIIGP.PDTLYDGG.YFNAIMT
Ta_Ubc7_#      1  .....MATAPARRASSSRSSSESRTT.PSMG.FQLGFVDDSNVFEWQVTIIGP.PETLYDGG.YFNAIMS
Vv_1_#         1  .....MASTTQACLLLQKQLRDLCKR.PVDG.FSAGLVDESNVFEWSVSIIGP.PDTLYDGG.FFNAIMS
Vv_2_#         1  ...MASASTNQASLLLQKQLKDLCKN.PVDG.FSAGLVDESNIFEWSVTIIGP.PDTLYDGG.FFNAIMS
Ah_#           1  .......MASQASLLLQKQLKDLCKN.PVDG.FSAGLVDESNIFEWSVTVIGP.PDTLYEGG.FFNAIMS
At_Ubc7_#      1  .......MASQASLLLQKQLKDLCKH.PVDG.FSAGLVDEKNIFEWSVTIIGP.PDTLYEGG.FFNAIMT
At_Ubc13_#     1  .......MNSQACLLLQKQLKDLCKH.PVDG.FSAGLVDEKNIFEWSVTIIGP.PDTLYEGG.FFYAIMS
At_Ubc14_#     1  ......MANNQASLLLQKQLKDLCKK.PVDG.FSAGLVDEKNVFQWSVSIMGP.PDTLYEGG.FFNAIMS
St_#           1  .......MASQASLLLQKQLKDLCKH.PVDG.FSAGLVDESNLFEWSVTIIGP.QDTLYEGG.FFNAIMS
Pt_#           1  ......MAASQAILLLQKQLRDLCKN.PVDG.FSAGLVDETDMFEWSVSIIGP.PDTLYDGG.FFNAIMS
Pp_1_#         1  ......MAENQASLLLRKQLKDLSRN.PVDG.FSAGLVDDSSVFEWNVTIIGP.SETLYEGG.FFNAIMS
Pp_2_#         1  ......MAENQASLLLRKQLKDLSRN.PVDG.FSAGLVDDSNLFEWNVTIIGP.PDTLYEGG.YFTAIMS
Psi_1_#        1  ......MGDFQAILLLRKQLKELSRH.PLDG.FSAGLVDDSNVFEWNVTIIGP.PDTIYEGG.YYNAIMT
Psi_2_#        1  ......MGENQASLLLRKQLKELSRN.PVDG.FSAGLVDDSNLFEWNVAIMGP.PDTLYEGG.YFNAIMS
Cr_#           1  ......MTDALAASLLRKQLKELTRN.PVDG.FSAGLVDDSNVFEWQVTIIGP.PETLYEGG.FFNARLT
Ol_#           1  .......MSDQAVLLLRKQLKELSRN.PIEG.FSAGLVDDCNVFEWQVTVMGP.PETLYEGG.FFTCNMS
Hs_Ube2R1      1  ...MARPLVPSSQKALLLELKGLQEE.PVEG.FRVTLVDEGDLYNWEVAIFGP.PNTYYEGG.YFKARLK
Mm_Ube2R1      1  ...MARPLVPSSQKALLLELKGLQEE.PVEG.FRVTLVDEGDLYNWEVAIFGP.PNTYYEGG.YFKARLK
Rn_R1          1  ...MARPLVPSSQKALLLELKGLQEE.PVEG.FRVTLVDEGDLYNWEVAIFGP.PNTYYEGG.YFKARLK
Md_R1          1  ...MARPLVPSSQKALLLELKGLQEE.PVEG.FRVTLVDEGDLYNWEVAIFGP.PNTYYEGG.YFKARLK
Gg_R1          1  ...MARSLVPSSQKALLLELKGLQEE.PVEG.FRVNLVDEGDLYNWEVAIFGP.PNTYYEGG.YFKARLR
Xl_R1          1  .MARPMAPVPSSQKALMLELKGLQEE.PVEG.FRVTLVGEGDLYNWEVAIFGP.PNTLYEGG.YFKARLK
Xt_R1          1  .MARPVAPVPSSQKALMLELKGLQEE.PVEG.FRVTLVDEGDLYNWEVAIFGP.PNTLYEGG.YFKARLK
Hs_Ube2R2      1  ...MAQQQMTSSQKALMLELKSLQEE.PVEG.FRITLVDESDLYNWEVAIFGP.PNTLYEGG.YFKAHIK
Mm_Ube2R2      1  ...MAQQQMTSSQKALMLELKSLQEE.PVEG.FRITLVDESDLYNWEVAIFGP.PNTLYEGG.YFKAHIK
Rn_R2          1  ...MAQQQMTSSQKALMLELKSLQEE.PVEG.FRITLVDESDLYNWEVAIFGP.PNTLYEGG.YFKIHLR
Md_R2          1  ...MAQQQMTSSQKALMLELKSLQEE.PVEG.FRITLVDESDLYNWEVAIFGP.PNTLYEGG.YFKAHIK
Gg_R2          1  ....MAQQQMSSQKALMLELKSLQEE.PVEG.FRITLVDESDLYNWEVAIFGP.PNTLYEGG.YSKAHIK
Xl_R2          1  ...MAHQQMTSSQKALMLELKSLQEE.PVEG.FRITLVDESDLYNWEVAIFGP.PNTLYEGG.YFKAHIK
Xt_R2          1  ...MAQQQMTSSQKALMLELKSLQEE.PVEG.FRITLVDESDLYNWEVAIFGP.PNTLYEGG.YFKAHIK
Dr_R2          1  ...MAHQQMPSSQKALMLELKSLQEE.PVEG.FRITLVEESDLYNWEVAIFGP.PNTLYEGG.YFKAHIK
Tn_R2          1  ...MAHQATPSSQKALMMELKSLQEQ.PLEG.FRITLVTESDLYNWQVAIFGP.PNTLYEGG.YFKAHIK
Nv_R           1  ....MAQPTSSALRALALEYKSLQEE.PVEG.FRVKLVNEDNMFEWEVAIFGP.PDTLYQGG.YFKAHMK
Dm_CG7656      1  AAAPTTTPSSSAVRALAMEYKSLQEE.PVEG.FRVKLINDDNLFEWEVAIFGP.PDTLYQGG.YFKAHMK
Aa_R           1  ...MSSQPSSSAVRALSLEYKSLQEE.PVEG.FRVKLLNEDNLFEWEVAIFGP.PDTLYQGG.YFKAHMK
Tc_R           1  ..MATTKATSSALRALAVEYKSLQEE.PVEG.FCVKLVNEDNLFEWEVAIFGP.PDTLYMGG.CFKARMK
Ce_Ubc3        1  .MDSKASTSSGALRALTMELKNLQSQ.PVEG.FTID.VNEDNLFVWTVGIYGP.PKTLYQGG.YFKASIR
Cb_R           1  .MDSRASTSSGAMRALSMELKNLQSQ.PVEG.FTID.VNEDNMFLWSVGIYGP.PMTLYQGG.YFKATIR
Cp_R           1  ...MSSQPSSSAVRALSLEYKSLQEE.PVEG.FRVKLLNEDNLFEWEVAIFGP.PDTLYQGG.YFKAHMK
Am_R           1  ....MAQTTSSALRALALEYKSLQEE.PVEG.FRVKLVNEDNMFEWEVAIFGP.PDTLYQGG.YFKAHMK
Sc_Ubc3        1  ....MSSRKSTASSLLLRQYRELTDPKKAIPSFHIELEDDSNIFTWNIGVMVLNEDSIYHGG.FFKAQMR
Cg_R           1  ....MSNLKSTAASLLLRQYRELTDPKKAIPSFHIELEDDSNIFVWNIGVMVLNEDSIYHGG.YFKAQMR
Vp_R           1  ....MSNRKSTAASLLLRQYRELTNPKKAIPSFHINLEDDSNIFVWNIGVMVLNEDSIYHGG.YFKAQMR
Asg_R          1  ....MSNRKNTAASLLLRQYRELTDPKKAIPSFHIELDDDSNIFSWNIGVMVLNEDSIYHGG.YFKAQMR
Pg_R           1  ......MSSKSAAAILQRQFKDLTDPKKGIPSFHIEL.DDDNIFLWNIGIMVLNKESMYHGG.YFKGQMR
Dh_R           1  ......MSSKSAAAILQRQFKDLTDPKKGIPSFHIEL.DDDNIFLWNIGIMVLNKESMYHGG.YFKGQMR
Ps_R           1  ......MSSKSAAAILQRQFKELTDPKKAIPSFHIEL.DDDNIFLWNIGIMVLNKDSMYHGG.YFKGQMR
Ca_R           1  ......MSSKSAAAILQRQFKDLTDPKKGIPSFHIEL.DDDNIFLWNVGVMVLNPESLYHGG.YFKGQMR
Le_R           1  ......MSSKSAAAILQRQFKDLTDPKKRIPSFHIEL.DDDNIFLWNIGVMVLNPDSLYHGG.YFKGQMR
```

```
                            β4       TT    TT   TT       TT   TT    TT   TT                 α2
Hs_Ube2G2
Hs_Ube2G2     60  FPLDYPLSPPKMRFTCEMFHPNIYPDGRVCISILHAPGDDPMGYESSAERWSPVQSVEKILLSVVSMLAE
Mm_Ube2G2     60  FPLDYPLSPPKMRFTCEMFHPNIYPDGRVCISILHAPGDDPMGYESSAERWSPVQSVEKILLSVVSMLAE
Rn_G2         60  FPLDYPLSPPKMRFTCEMFHPNIYPDGRVCISILHAPGDDPMGYESSAERWSPVQSVEKILLSVVSMLAE
Md_G2         60  FPLDYPLSPPKMRFTCEMFHPNIYPDGRVCISILHAPGDDPMGYESSAERWSPVQSVEKILLSVVSMLAE
Gg_G2         60  FPLDYPLSPPKMRFTCEMFHPNIYPDGRVCISILHAPGDDPMGYESSAERWSPVQSVEKILLSVVSMLAE
Xl_G2         60  FPLDYPLSPPKMRFTCEMFHPNIYPDGRVCISILHAPGDDPMGYESSAERWSPVQSVEKILLSVVSMLAE
Xt_G2         60  FPLDYPLSPPKMRFTCEMFHPNIYPDGRVCISILHAPGDDPMGYESSAERWSPVQSVEKILLSVVSMLAE
Dr_G2         60  SPSDYPPSPPKMKFTCDMFHPNIYPDGRVCISILHAPGDDPMGYESSAERWSPVQSVEKILLSVVSMLAE
Tn_G2         60  FPSDYPLSPPKMRFTCEMFHPNIYPDGRVCISILHAPGDDPMGYESSTERWSPVQSVEKILLSVVSMLAE
Nv_G2         60  FPTDYPLSPPKMQFTCEIFHPNIYSDGRVCISILHAPGDDPMGYESSAERWSPVQSVEKILLSVVSMLAE
Dm_G2         60  FPTDYPLSPPKMKFTCDMFHPNIFADGRVCISILHAPGDDPMGYELSAERWSPVQSVEKILLSVVSMLAE
Dp_G2         60  FPPDYPLSPPKMKFTCDMFHPNIFADGRVCISILHAPGDDPLGYELSAERWSPVQSVEKILLSVVSMLAE
Aa_G2         60  FPPDYPLSPPKMRFTCEMFHPNIFADGRVCISILHAPGDDPLGYELSAERWSPVQSVEKILLSVVSMLAE
Tc_G2         60  FPPDYPLSPPKMQFTCEMFHPNIYADGRVCISILHAPGDDPMGYESSAERWSPVQSVEKILLSVVSMLAE
Ce_Ubc14      60  FPQDYPLSPPKMRFTCGIFHPNIYADGRVCISILHAPGDDPTGYELSNERWSPVQSIEKILLSVVSMLAE
Cb_G2         60  FPQDYPLSPPKMRFTCGIFHPNIYPDGRVCISILHAPGDDPTGYESSNERWSPVQSIEKILLSVVSMLAE
Cp_G2         60  FPPDYPLSPPKMRFTCEMFHPNIFADGRVCISILHAPGDDPLGYELSAERWSPVQSVEKILLSVVSMLAE
Am_G2         60  FPPDYPLSPPKMQFTCEMFHPNIYADGRVCISILHAPGDDPMGYESSAERWSPVQSVEKILLSVVSMLAE
Sp_Ubc3       61  FPSDYPLGPPTLKFECEFFHPNVYKDGTVCISILHAPGDDPNMYESSSERWSPVQSVEKILLSVMSMLAE
Sc_Ubc7       60  FPKDYPLSPPKLTFTPSILHPNIYPNGEVCISILHSPGDDPNMYELAEERWSPVQSVEKILLSVMSMLSE
Cg_G2         60  FPKDYPLSPPKLTFTPSILHPNIYPNGEVCISILHSPGDDPNMYESAEERWSPVQSVEKILLSVMSMLSE
Vp_G2         60  FPRDYPLSPPTLTFTPAILHPNIYPNGEVCISILHSPGDDPNMYELAEERWSPVQSVEKILLSVMSMLSE
Asg_G2        60  FPRDYPLSPPKLTFTPSILHPNVYPNGEVCISILHAPGEDPNMYEEASERWSPVQSVEKILLSVMSMLSE
Pg_G2         61  FPKDYPLSPPVLKFDPPILHPNVYADGTVCISILHAPGEDPNHYERPEERWSPVQSIEKILLSVVSMLAE
Dh_G2         62  FPKDYPLSPPTLTFDPPLLHPNIYADGTVCISILHAPGEDPNQYERPEERWSPVQSIEKILLSVMSMLAE
Ps_G2         62  FPKDYPLSPPSLKFDPPLLHPNIYADGTVCISILHPSGEDPNQYERPEERWSPVQSIEKILLSVMSMLAE
Ca_G2         62  FPKDYPLSPPTLKFDPPLLHPNIYADGTVCISILHPPGEDPNQYERPEERWSPVQSIEKILLSVMSMLAE
Le_G2         62  FPKDYPLGPPTLKFDPPLLHPNIYADGTVCISILHPPGEDPNQYERPEERWSPVQSIEKILLSVISMLAE
Hs_Ube2G1     61  FPKDYPLRPPKMKFITEIWHPNVDKNGDVCISILHEPGEDKYGYEKPEERWLPIHTVETIMISVISMLAD
Mm_Ube2G1     61  FPKDYPLRPPKMKFITEIWHPNVDKNGDVCISILHEPGEDKYGYEKPEERWLPIHTVETIMISVISMLAD
Rn_Ube2G1     61  FPKDYPLRPPKMKFITEIWHPNVDKNGDVCISILHEPGEDKYGYEKPEERWLPIHTVETIMISVISMLAD
Md_G1         61  FPKDYPLRPPEMKFITEIWHPNVDKNGDVCISILHEPGEDKYGYEKPEERWLPIHTVETIMISVISMLAD
Gg_G1         61  FPKDYPLRPPKMKFITEIWHPNVDKNGDVCISILHEPGEDKYGYEKPEERWLPIHTVETIMISVISMLAD
Xl_G1         61  FPRDYPLRPPKMKFITEIWHPNVDKNGDVCISILHEPGEDKYGYEKPEERWLPIHTVETIMISVISMLAD
Xt_G1         61  FPRDYPLRPPKMKFITEIWHPNVDKNGDVCISILHEPGEDKYGYEKPEERWLPIHTVETIMISVISMLAD
Dr_1_G1       61  FPKDYPLRPPKMKFITEIWHPNVDKNGDVCISILHEPGEDKYGYEKPEERWLPIHTVETIMISVISMLAD
Dr_2_G1       60  FPHDYPLRPPKMKFITEIWHPNVAKNGDVCISILHEPGEDKFGYEKPEERWLPIHTVETIMISVISMLAD
Tn_1_G1       60  FPSDYPLRPPKMKFISQMWHPNVAKNGEVCISILHEPGEDKYGYERPEERWLPIHTVETIMISVISMLAD
Tn_2_G1       62  FPKDYPLRPPKMKFITDIWHPNVDKNGDVCISILHEPGEDKYGYEKPEERWLPIHTVETIMISVISMLAD
Nv_G1         61  FPKEYPLRPPRMKFITEIWHPNIEKNGDVCISILHEPGDDKWGYEKASERWLPVHTVETILISVISMLAD
Dm_CG40045    61  FPKEYPLRPPRMKFVTEIWHPNIEKNGDVCISILHEPGDDKWGYEKASERWLPVHTVETILISVISMLAD
Dm_CG9602     61  FPKEYPLRPPKMKFITEIWHPNIDKAGDVCISILHEPGDDKWGYEKAEERWLPVHTVETILLSVISMLTD
Dp_G1         61  FPKEYPLRPPTMKFVTDIWHPNIEKGGNVCISILHEPGDDKWGYEKAEERWLPVHTVETILLSVISMLTD
Aa_G1         61  FPKEYPLRPPRMKFVTEIWHPNIDRNGDVCISILHEPGDDKWGYEKASERWLPVHTVETILISVISMLAD
Tc_G1         61  FPKEYPLRPPKMKFVTEIWHPNIDKNGDVCISILHEPGDDKFGYEKASERWLPVHTVETILISVISMLAD
Ce_Ubc7       59  FPRDYPQKPPKMKFISEIWHPNIDKEGNVCISILHDPGDDKWGYERPEERWLPVHTVETILLSVISMLTD
Cb_G1         59  FPRDYPQKPPKMKFISEIWHPNIDKEGNVCISILHDPGDDKWGYERPEERWLPVHTVETILLSVISMLTD
Sp_Ubc15      61  FPQDYPLMPPKMKFTTEIWHPNVHPNGEVCISILHPPGDDKYGYEDAGERWLPVHSPETILISVISMLSS
Os_1_#        63  FPQNYPNSPPSVRFTSEMWHPNVYPDGRVCISILHPPGEDPNGYELASERWTPVHTVESIVLSIISMLSS
Os_2_#        63  FPQNYPNSPPTVRFTSEMWHPNVYPDGRVCISILHPPGDDPNGYELASERWTPVHTVESIVLSIISMLSG
Zm_1_#        63  FPQNYPNSPPSVRFTSEMWHPNVYPDGRVCISILHPPGEDPNGYELASERWTPVHTVESIVLSIISMLSS
Zm_2_#        63  FPQNYPNSPPSVRFTSEMWHPNVYPDGRVCISILHPPGEDPNGYELASERWTPVHTVESIVLSIISMLSS
Ta_Ubc7_#     62  FPQNYPNSPPTVRFTSEMWHPNVYPDGRVCIS.IHPPGDDPNGYELASERWTPVHTVESIVLSIISMLSS
Vv_1_#        62  FPPNYPNSPPSVRFTSDMWHPNVYPDGRVCISILHPPGEDPNGYELASERWTPVHTVESIVLSIISMLSS
Vv_2_#        64  FPPNYPNSPPTVKFTSEVWHPNVYPDGRVCISILHPPGEDPNGYELASERWMPIHTVESIVLSIISMLSS
Ah_#          60  FPSNYPNSPPTVKFTSEIWHPNVYPDGRVCISILHPPGDDPNGYELASERWNPVHTVESIVLSIISMLSS
At_Ubc7_#     60  FPQNYPNSPPTVRFTSDMWHPNVYSDGRVCISILHPPGDDPSGYELASERWTPVHTVESIMLSIISMLSG
At_Ubc13_#    60  FPQNYPNSPPTVRFTSDIWHPNVYPDGRVCISILHPPGDDPSGYELASERWTPVHTVESIMLSIISMLSG
At_Ubc14_#    61  FPENYPVSPPTVTFTSEMWHPNVYSDGKVCISILHPPGDDPHGYELASERWTPVHTVESIVLSIISMLSG
St_#          60  FPENYPNSPPTVRFTTEIWHPNVYSDGKVCISILHPPGDDPNGYEYASERWSPVHTVESIILSIISMLSS
Pt_#          61  FPKNYPNSPPTVRFTSEMWHPNVYPDGKVCISILHPPGDDPNGYELATERWSPVHTVESIVLSIISMLSS
Pp_1_#        61  FPPDYPNSPPTVRFTSEMWHPNVYPDGRVCISILHAPGDDPNGYELASERWSPVHTVETILISIIAMLSN
Pp_2_#        61  FPPNYPNSPPSVRFTSDMWHPNVYPDGRVCISILHAPGDDPNGYELASERWSPVHTVETILLSIISMLSS
Psi_1_#       61  FPPNYPNSPPTVRFTSEMWHPNVYPDGRVCISILHPPGDDPNGYELASERWTPVHTVETILLSIISMLSS
Psi_2_#       61  FPPNYPNSPPSVRFTSEMWHPNVYPDGRVCISILHAPGDDPNGYELASERWSPVHTVETILLSIISMLSS
Cr_#          61  FPKDYPNSPPTCRFTSEMWHPNVYDDGKVCISILHSPGDDPSGYETAAERWSPVHTVETIMISIISMLSS
Ol_#          60  FPKDYPNKPPTVKFVSEMWHPNVYADGRVCISILHSPGNDPNGYEDASERWSPVQTVETIMLSVISMLSD
Hs_Ube2R1     64  FPIDYPYSPPAFRFLTKMWHPNIYETGDVCISILHPPVDDPQSGELPSERWNPTQNVRTILLSVISLLNE
Mm_Ube2R1     64  FPIDYPYSPPAFRFLTKMWHPNIYETGDVCISILHPPVDDPQSGELPSERWNPTQNVRTILLSVISLLNE
Rn_R1         64  FPIDYPYSPPAFRFLTKMWHPNIYETGDVCISILHPPVDDPQSGELPSERWNPTQNVRTILLSVISLLNE
Md_R1         64  FPIDYPYSPPAFRFLTKMWHPNIYETGDVCISILHPPVDDPQSGELPSERWNPTQNVRTILLSVISLLNE
Gg_R1         64  FPIDYPYSPPAFRFLTKMWHPNIYETGDVCISILHPPVDDPQSGELPSERWNPTQNVRTILLSVISLLNE
Xl_R1         66  FPVDYPYSPPAFRFLTKMWHPNIYENGDVCISILHPPVDDPQSGELPSERWNPTQNVRTILLSVISLLNE
Xt_R1         66  FPVDYPYSPPAFRFLTKMWHPNIYENGDVCISILHPPVDDPQSGELPSERWNPTQNVRTILLSVISLLNE
Hs_Ube2R2     64  FPIDYPYSPPTFRFLTKMWHPNIYENGDVCISILHPPVDDPQSGELPSERWNPTQNVRTILLSVISLLNE
Mm_Ube2R2     64  FPIDYPYSPPTFRFLTKMWHPNIYENGDVCISILHPPVDDPQSGELPSERWNPTQNVRTILLSVISLLNE
Rn_R2         64  LPPDYRCVPPGLAFCGFVWHPNIYENGDVCISILHPPVDDPQSGELPSERWNPTQNVRTILLSVISLLNE
Md_R2         64  FPIDYPYSPPTFRFLTKMWHPNIYENGDVCISILHPPVDDPQSGELPSERWNPTQNVRTILLSVISLLNE
Gg_R2         63  FPIDYPYSPPTFRFLTKMWHPNIYENGDVCISILHPPVDDPQSGELPSERWNPTQNVRTILLSVISLLNE
Xl_R2         64  FPIDYPYSPPTFRFLTKMWHPNIYENGDVCISILHPPVDDPQSGELPSERWNPTQNVRTILLSVISLLNE
Xt_R2         64  FPIDYPYSPPTFRFLTKMWHPNIYENGDVCISILHPPVDDPQSGELPSERWNPTQNVRTILLSVISLLNE
Dr_R2         64  FPIDYPYSPPTFRFLTKMWHPNIYENGDVCISILHPPVDDPQSGELPSERWNPTQNVRTILLSVISLLNE
Tn_R2         64  FPVDYPYSPPTFRFLTKMWHPNIYENGDVCISILHPPVDDPQSGELPSERWNPTQNVRTILLSVISLLNE
Nv_R          63  FPPDYPYSPPSIRFMTKVWHPNVYENGDLCISILHPPVDDPQSGELPCERWNPTQNVRTILLSVISLLNE
Dm_CG7656     67  FPHDYPYSPPSIRFLTKVWHPNVYENGDLCISILHPPVDDPQSGELPCERWNPTQNVRTILLSVISLLNE
Aa_R          64  FPPDYPYSPPSIRFLTKVWHPNVYENGDLCISILHPPVDDPQSGELPCERWNPTQNVRTILLSVISLLNE
Tc_R          65  FPPDYPYSPPSIRFLTKVWHPNVYENGDLCISILHPPVDDPQSGELPCERWNPTQSVRTILLSVISLLNE
Ce_Ubc3       65  FPSNYPYSPPSMKFTTKVWHPNVYENGDLCISILHSPIDDPQSGELACERWNPTQSVRTILLSVISLLNE
Cb_R          65  FPSNYPYQPPSMKFTTKVWHPNVYENGDLCISILHAPVDDPQSGELACERWNPTQSVRTILLSVISLLNE
Cp_R          64  FPPDYPYSPPSIRFLTKVWHPNVYENGDLCISILHPPVDDPQSGELPCERWNPTQNVRTILLSVISLLNE
Am_R          63  FPPDYPYSPPSIRFMTKVWHPNVYENGDLCISILHPPVDDPQSGELPCERWNPTQNVRTILLSVISLLNE
Sc_Ubc3       66  FPEDFPFSPPQFRFTPAIYHPNVYRDGRLCISILHQSG.DPMTDEPDAETWSPVQTVESVLISIVSLLED
Cg_R          66  FPDDFPFSPPQFRFTPAIYHPNVYRDGRLCISILHQSG.DPMTDEPDAETWSPVQTVESVLISIVSLLED
Vp_R          66  FPDDFPFSPPQFRFTPAIYHPNVYRDGRLCISILHQSG.DPMTDELDAETWSPVQTVESVLISIVSLLED
Asg_R         66  FPEDFPFSPPSFRFTPAIYHPNVYRDGRLCISILHQSG.DPTSDEPDSETWSPVQTVESVLISIVSLLED
Pg_R          63  FPSEFPFAPPTFRFTPAIYHPNVYRDGRLCISILHQGG.DPTSDEPDSETWTPAQTVELVLISIISLLED
Dh_R          63  FPSEFPFSPPTFRFTPAIYHPNVYRDGRLCISILHQGG.DPTSDEPDSETWTPAQTVESVLISIISLLED
Ps_R          63  FPSDFPFAPPTFRFTPAIYHPNVYRDGRLCISILHQGG.DPTSDEPDSETWTPAQTVESVLISIVSLLED
Ca_R          63  FPQDFPFSPPSFRFTPALYHPNVYRDGRLCISILHQGG.DPTSDEPESETWSPAQTVESVLISIISLLDD
Le_R          63  FPQDFPFSPPSFRFTPAIYHPNIYRDGRLCISILHQGG.DPTSDEPESETWSPAQTVESALISIISLLDD
```

```
                          α3                    α4
Hs_Ube2G2         TT   ℓℓℓℓℓℓℓℓ       ℓℓℓℓℓℓℓℓℓℓℓℓℓℓℓℓ
Hs_Ube2G2     130 PNDESGANVDASKMWRD.......DREQFYKIAKQIVQKSLGL
Mm_Ube2G2     130 PNDESGANVDASKMWRD.......DREQFYKIAKQIVQKSLGL
Rn_G2         130 PNDESGANVDASKMWRD.......DREQFYKIAKQIVQKSLGL
Md_G2         130 PNDESGANVDASKMWRD.......DREQFYRIAKQTVQKSLGL
Gg_G2         130 PNDESGANVDASKMWRE.......DREQFNKIAKQIVQKSLGL
Xl_G2         130 PNDESGANVDASKMWRE.......DREQFNTIARQTVQKSLGL
Xt_G2         130 PNDESGANVDASKMWRE.......DREQFNKIARQTVQKSLGL
Dr_G2         130 PNDESGANVDASKMWRE.......DREQFNRLAKQIVRKSLGL
Tn_G2         130 PNDESGANVDASKMWRE.......DRDQFNKLAHKIVRKSLGL
Nv_G2         130 PNDESGANVDAAKMWRE.......DRTEFERIAQKLVRKTLGI
Dm_G2         130 PNDESGANVDAAIMWRE.......QRDEFNAIARRLVRKTLGL
Dp_G2         130 PNDESGANVDAAIMWRE.......RREEFNSIARRLVRKTLGL
Aa_G2         130 PNDESGANVDAAIMWRE.......NREEFNKIAKRIVRKTLGL
Tc_G2         130 PNDESGANVDAAKMWRE.......NREEFNRIAERIVRRTLGI
Ce_Ubc14      130 PNDESPANVSAAKMWRE.......DRAQFEKIADSLVRKTLCL
Cb_G2         130 PNDESPANVNAAKMWRE.......DRAQFEKTADALVRKTLCL
Cp_G2         130 PNDESGANVDAAIMWRE.......NRDEFNKIAKRIVRKSLGL
Am_G2         130 PNDESGANVDAAKMWRE.......DRSEFERIAQKLVRKTLGI
Sp_Ubc3       131 PNDESGANIDACKMWRE.......DREEYCRVVRRLARKTLGL
Sc_Ubc7       130 PNIESGANIDACILWRD.......NRPEFERQVKLSILKSLGF
Cg_G2         130 PNIESGANIDACILWRD.......NRAEFEKQVKLSILKSLGF
Vp_G2         130 PNVESGANIDACILWRD.......NRPEFERQVKRSIVASLGL
Asg_G2        130 PNVESGANIDACILWRD.......NRADFERQVQESVRRSLGL
Pg_G2         131 PNPESGANVDACKLWRD.......HRQEFDRQVRSHVRQSLGL
Dh_G2         132 PNPESGANIDACKLWRD.......NRVEYDRQIRDHVRKTLGL
Ps_G2         132 PNPESGANIDACKLWRD.......NRAEYNSQIKKHVRESLGL
Ca_G2         132 PNPESGANIDACKLWRD.......NRAEYDRQIRQHVKESLGL
Le_G2         132 PNPESGANIDACKLWRD.......NRELFDEQVGSHVRKSLGL
Hs_Ube2G1     131 PNGDSPANVDAAKEWRE......DRNGEFKRKVARCVRKSQET
Mm_Ube2G1     131 PNGDSPANVDAAKEWRE......DRNGEFKRKVARCVRKSQET
Rn_Ube2G1     131 PNGDSPANVDAAKEWRE......DRNGEFKRKVARCVRKSQET
Md_G1         131 PNGDSPANVDAAKEWRE......DRNGEFKRKVARCVRRSQET
Gg_G1         131 PNGDSPANVDAAKEWRE......DRNGEFKRKVARCVRKSQET
Xl_G1         131 PNGDSPANVDAAKEWRE......DRNGEFKRKVARCVRKSQET
Xt_G1         131 PNGDSPANVDAAKEWRE......DRNGEFKRKVARCVRKSQET
Dr_1_G1       131 PNGDSPANVDAAKEWRE......DRHGEFKRKVARCVRKSQET
Dr_2_G1       130 PNGDSPANVDAAKEWRD......DPNGEFKRKVARCVRKSQEM
Tn_1_G1       130 PNGDSPANVDAAKEWRE......DPQGVFKRKVARCVRRSQEE
Tn_2_G1       132 PNGDSPANVDAAKEWRE......DRDGEFKRKVARCVRKSQD.
Nv_G1         131 PNDESPANVDAAKEWRE.......SYTEFKRKVARCVRKSQEE
Dm_CG40045    131 PNDESPANVDAAKEWRE.......SYTDFKRKVARCVRKSQEE
Dm_CG9602     131 PNDESAANVDAAKEYRE.......NYAEFKRKVTRCVRRSQEE
Dp_G1         131 PNSESAANVDAAKEYRE.......DPAEFKRKVARCVRRSQEE
Aa_G1         131 PNDESPANVDAAKEWRE.......AYPEFKRKVARCVRKSQED
Tc_G1         131 PNDESPANVDAAKEWRE.......MYGEFKRKVARCVRKSQEE
Ce_Ubc7       129 PNFESPANVDAAKMQRE.......NYAEFKKKVAQCVRRSQEE
Cb_G1         129 PNFESPANVDAAKMQRE.......NYADFKKKVAQCVRRTQEE
Sp_Ubc15      131 PNDESPANIDAAKEFRE.......NPQEFKKRVRRLVRRSIEM
Os_1_#        133 PNDESPANIEAAKDWRE.......KRDDFKKKVRRIVRKSQEM
Os_2_#        133 PNDESPANIEAAKEWRE.......KRDDFKKKVRRLVRKSQEM
Zm_1_#        133 PNDESPANIEAAKEWRE.......QREDFKKKVRRIVRKSQEM
Zm_2_#        133 PNDESPANIEAAKDWRE.......KRDEFKKKVRQCVRKSQEM
Ta_Ubc7_#     131 PNDESPANIEAAKDWRE.......KQDEFKKKVRRAVRKSQEM
Vv_1_#        132 PNDESPANIEAAKEWRE.......KRDEFKKKVSRCVRKSQEM
Vv_2_#        134 PNDESPANIEAAKEWRD.......RRDEFKKKVSRCVRRSQEM
Ah_#          130 PNDESPANVEAAKEWRE.......RRDDFKKKVSRCVRKSQEM
At_Ubc7_#     130 PNDESPANVEAAKEWRD.......KRDEFKKKVSRCVRKSQEM
At_Ubc13_#    130 PNDESPANVEAAKEWRE.......KRDEFKKKVSRCVRKSQEM
At_Ubc14_#    131 PNDESPANVEAAKEWRD.......NRAEFRKKVSRCVRRSQEM
St_#          130 PNDESPANVEAAKEWRE.......KRDEFKKRVSRCVRRSQDL
Pt_#          131 PNDESPANVDAAKQWRE.......SREEFRKKVSRCVRKSQEM
Pp_1_#        131 PNDESPANVDAAKEWRE.......QRDEFQRKVRRIVRRSQEF
Pp_2_#        131 PNDESPANIDAAKEWRE.......TKEEFKRKVSRIVRRSQES
Psi_1_#       131 PNDESPANIDAAKEWRD.......HRDEFRKKVGRIVRKSQET
Psi_2_#       131 PNDESPANIDAAKEWRE.......HRDEFKKKVSRIVRRSQES
Cr_#          131 PNDESPANVDAAKMWRE.......NRDEFKKRVARIVRKSQEM
Ol_#          130 PNDESPANVDAAKEWRD.......DYPAFKKRVARCVRRSQEE
Hs_Ube2R1     134 PNTFSPANVDASVMYRKWKESKGKDR.EYTDIIRKQVLGTKVD
Mm_Ube2R1     134 PNTFSPANVDASVMYRKWKESKGKDR.EYTDIIRKQVLGTKVD
Rn_R1         134 PNTFSPANVDASVMYRKWKESKGKDR.EYTDIIRKQVLGTKVD
Md_R1         134 PNTFSPANVDASVMYRKWKDSKGKDR.EYTDIIRKQVLGTKVD
Gg_R1         134 PNTFSPANVDASVMYRKWKESKGKDR.EYTDIIRKQVLGTKVD
Xl_R1         136 PNTFSPANVDASVMYRKWKDSKGRDK.EYTDIIRKQVLSTNAD
Xt_R1         136 PNTFSPANVDASVMYRKWKDSKGRDK.EYTDIIRKQVVSTKAD
Hs_Ube2R2     134 PNTFSPANVDASVMFRKWRDSKGKDK.EYAEIIRKQVSATKAE
Mm_Ube2R2     134 PNTFSPANVDASVMFRKWRDSKGKDK.EYAEIIRKQVSATKAE
Rn_R2         134 PNTFSPANVDASVMFRKWRDSKGKDK.EYAEIIRKQVSATKAE
Md_R2         134 PNTFSPANVDASVMFRKWRDSKGKDK.EYAEIIRKQVLATKAE
Gg_R2         133 PNTFSPANVDASVMFRKWRDSKGKDK.EYAEIIRKQVLATKAE
Xl_R2         134 PNTFSPANVDASVMFRKWRDSKGKDK.EYAEIIRKQVSATKAE
Xt_R2         134 PNTFSPANVDASVMFRKWRDSKGKDK.EYAEIIRKQVSATKAE
Dr_R2         134 PNTFSPANVDASVMFRKWRDSKGKDK.EYAEIIRKQVVSTKAD
Tn_R2         134 PNTFSPANVDASVMFRKWRDSKGKDK.EYAEIIRKQVLSTAAE
Nv_R          133 PNTYSPANVDASVMYRRWRDSKGRDK.EYENIIRKQAQAAKVE
Dm_CG7656     137 PNTFSPANVDASVMYRRWRDSQGKDN.EYPNIIRKQALAANAE
Aa_R          134 PNTFSPANVDASVMYRRWRDSQGKDN.EYPNIIRKQAQAAKAE
Tc_R          135 PNTFSPANVDASVMYRRWRDSKGRDK.EYENIIRKQAMAARAE
Ce_Ubc3       135 PNTSSPANVDASVMYRKWKED..QDP.EYAKIVTKQVEESKKV
Cb_R          135 PNTSSPANVDASVMYRKWKEE..QDP.EYAKIVKKQVEDSKKV
Cp_R          134 PNTFSPANVDASVMYRRWRDSQGKDN.EYPNIIRKQAQAAKAE
Am_R          133 PNTYSPANVDASVMYRRWRDSKGKDK.EYENIIRKQAQAAKME
Sc_Ubc3       135 PNINSPANVDAAVDYRK.......NPEQYKQRVKMEVERSKQD
Cg_R          135 PNISSPANVDAAVDFRK.......NPEQYKQRVKMEVERSKQD
Vp_R          135 PNISSPANVDAAVDYRK.......NPEQYKQRVKMEVERSKQD
Asg_R         135 PNISSPANVDAAVDYRK.......NPEQYKQRVKLEVERSKQD
Pg_R          132 PNVLLPANIDASVELRK.......NPEAYKKRVLQEVERSKQD
Dh_R          132 PNVSSPANIDASVELRK.......NPDAYKKKVMQEVERSKAD
Ps_R          132 PNVSSPANIDASVEFRK.......NPEAYKKKVLHEVERSKAD
Ca_R          132 PNGNSPANIDASVEFRK.......NYNVYKKKVLQEVERSKKD
Le_R          132 PNGNSPANIDASVEFRK.......NPEEYKKKVLKEVERSKKD
```
