# Supplementary material for: Loop 7 of E2 Enzymes: An Ancestral Conserved Functional Motif Involved in the E2-Mediated Steps of the Ubiquitination Cascade
Source: PLoS One. 2012 Jul 18;7(7):e40786. doi: 10.1371/journal.pone.0040786 (PMC3399832; doi:10.1371/journal.pone.0040786)
Supplement: Table S2 — Consensus of chained correlations derived by NMA, BD and DMD methods, as implemented in FlexServ, using as root residues the hydrophobic residues of L7. (PDF) [file pone.0040786.s003.pdf]

	System	Loop 7	Loop 8		
			Depth		
			**3**	**4**	**5**
**EnsembleA**	CDC34_1PZV → ScUbc3<br>CDC34_2CYX → ScUbc3<br>CDC34_2UCZ → ScUbc3	LEU 93		PRO 134, ALA 135	ILE 131, VAL 137, ALA 140
		PRO 99			
		MET 100			
		PRO 104			PRO 134, ALA 135
		ALA 106			PRO 134, ALA 135
	**1PZV → CeUbc7**	LEU 92		PRO 134, ALA 135	ALA 139, ALA 140
		PRO 95			
		PRO 105			PRO 134, ALA 135
	**2CYX → hsUbe2g2xray**	LEU 93		ALA 136	VAL 138, ALA 140
		ALA 95			ALA 136
		PRO 96			
		PRO 100			
		MET 101			
		ALA 107			ALA 136
	**2UCZ → ScUbc7**	LEU 93		GLY 135, ALA 136	ILE 138, ALA 140
		PRO 96			GLY 135, ALA 136
		PRO 100			
		MET 102			
		LEU 105			GLY 135

	ALA 106			GLY 135
**3FSH → MmUbe2g2**	LEU 93		GLY 135, ALA 136	VAL 138, ALA 140, SER 141
	ALA 95			GLY 135, ALA 136
	PRO 96			GLY 135, ALA 136
	PRO 100			
	MET 101			
	ALA 107			GLY 135, ALA 136
**At_Ubc7.B99990003 → Model**	LEU 93	PRO 135, ALA 136	VAL 138	ALA 140
	PRO 95	PRO 135, ALA 136	VAL 138	ALA 140
	PRO 96		PRO 135, ALA 136	PRO 130, VAL 138
	GLY 97		PRO 135, ALA 136	PRO 130
	PRO 100			
	LEU 105			PRO 135, ALA 136
	ALA 106			
**Dm_CG40045.B99990001 → Model**	LEU 94	PRO 136, ALA 137	VAL 139	PRO 131
	PRO 97		PRO 136, ALA 137	PRO 131, VAL 139
	GLY 98		PRO 136, ALA 137	PRO 131, VAL 139
	GLY 103			PRO 136, ALA 137
	ALA 107			PRO 136, ALA 137
**2OB4 → Hcdc34_Model 2**	PRO 91		PRO 131, ALA 132	VAL 134, ALA 136, VAL 138, MET 139
	PRO 92			PRO 131, ALA 132, SER 137
	VAL 93			PRO 131, ALA 132
	PRO 96			PRO 131, ALA 132, VAL 134, VAL 138
	GLY 99			VAL 134, ALA 136

	LEU 101		ALA 132, VAL 134	PHE 129, PRO 131, ALA 136, VAL 138, MET 139
	PRO 102		ALA 132, VAL 134	PHE 129, PRO 131, ALA 136, VAL 138, MET 139
**2OB4 → Hcdc34_Model 3**	PRO 91	PHE 129	PRO 131, ALA 132, VAL 134, ALA 136, VAL 138	MET 139
	PRO 92		PHE 129, SER 130, PRO 131	ALA 132, VAL 134, ALA 136, VAL 138, MET 139
	VAL 93			PHE 129, PRO 130, PRO 131, ALA 132, VAL 134, VAL 138
	PRO 96			
	LEU 101			PRO 131, ALA 132, VAL 134, ALA 136, VAL 138, MET 139
	PRO 102			
**Sp_Ubc3.B99990003 → Model**	LEU 94	GLY 136, ALA 137	PRO 131, ILE 139	ALA 129
	ALA 96		ALA 137	PRO 131. ILE 139, ALA 140
	PRO 97		GLY 136, ALA 137	PRO 131, ILE 139
	GLY 98		GLY 136, ALA 137	PRO 131, ILE 139
	PRO 101			
	MET 103			
**2KLY_c1 → hsUbe2g2NMR**	LEU 93		GLY 135, VAL 138	PRO 130
	ALA 95		ALA 136	GLY 135, VAL 138, ALA 140
	PRO 96			GLY 135, ALA 136
	PRO 100			
	MET 101			
	ALA 107			GLY 135, ALA 136
**2KLY_c4 → hsUbe2g2NMR**	LEU 93		GLY 135, ALA 136	VAL 138, ALA 140

	ALA 95		GLY 135	ALA 136, VAL 138
	PRO 96		GLY 135	ALA 136, VAL 138
	PRO 100		VAL 138	PRO 130
	MET 101			GLY 135
	ALA 107		GLY 135	ALA 136, VAL 138
**2KLY_c7 → hsUbe2g2NMR**	LEU 93			
	ALA 95			
	PRO 96			
	PRO 100		GLY 135, ALA 136	VAL 138, ALA 140
	MET 101		PRO 130, GLY 135, ALA 136	VAL 138, ALA 140
	ALA 107			PRO 130, GLY 135, ALA 136
