# Supplementary material for: Loop 7 of E2 Enzymes: An Ancestral Conserved Functional Motif Involved in the E2-Mediated Steps of the Ubiquitination Cascade
Source: PLoS One. 2012 Jul 18;7(7):e40786. doi: 10.1371/journal.pone.0040786 (PMC3399832; doi:10.1371/journal.pone.0040786)
Supplement: Text S1 — This file contains the following supporting figures for this article: Figure 2 . B-factor profiles obtained for the E2 representative members of family 3 by NMA, BD and DMD. Figure 3. Rmsf profiles from MD simulations. The 3D structure of each E2 family 3 enzyme is used as a reference and each residue colored with different shade of colors according to Cα rmsf values calculated from the MD simulations (from light to dark colors for increasing rmsf values). Text 1. Modelling of the E2-Ub complexes. Figure 4 . Ubiquitin position and orientation is shown to be different in the three known complexes with E2 enzymes (PDB code 1FXT, 3A33, 2KJH). Figure 5 . (A) E1 catalytic subunit SAE2 and Ubc9 have been crystallized in a structure with PDB code 2PX9 and it is the E1 structurally more similar to the Uba1 E1 enzymes for Ub. (B/C/D) Different ubiquitin positions have been obtained from the three known E2-Ub complexes through structural superimpositions. The only orientation that satisfies the spatial restraints imposed by the E1 structure is the one that appears in 1FXT E2-Ub complex (B). Figure 6 . (A) UBA3 (E1) and Ubc12 have been crystallized in a structure with PDB code 2NVU. (B/C/D) Different ubiquitin positions have been obtained from the three known E2-Ub complexes through structural superimpositions. The only orientation that satisfies the spatial restraints imposed by the E1 structure is the one that appears in 1FXT E2-Ub complex (B). Figure 7 . A preliminary model of an E1–E2 (family 3)-Ub complex has been obtained by superposing UBA1 structure (PDB code: 3CMM) to SAE2 structure (PDB code: 2PX9), using ubiquitin position as it appears in the complex Ubc1-Ub (1FXT). The catalytic cysteine of the E2 enzyme is indicated as yellow sphere and the E1 catalytic cysteine as yellow stick. Acidic L7 and phospho-S130 residues are shown as red spheres and the positively charged residues of Ub C-terminal chain as blue spheres. (PDF) [file pone.0040786.s004.pdf]

Figure 2. B-factor profiles obtained for the E2 representative members of family 3 by NMA, BD and DMD.

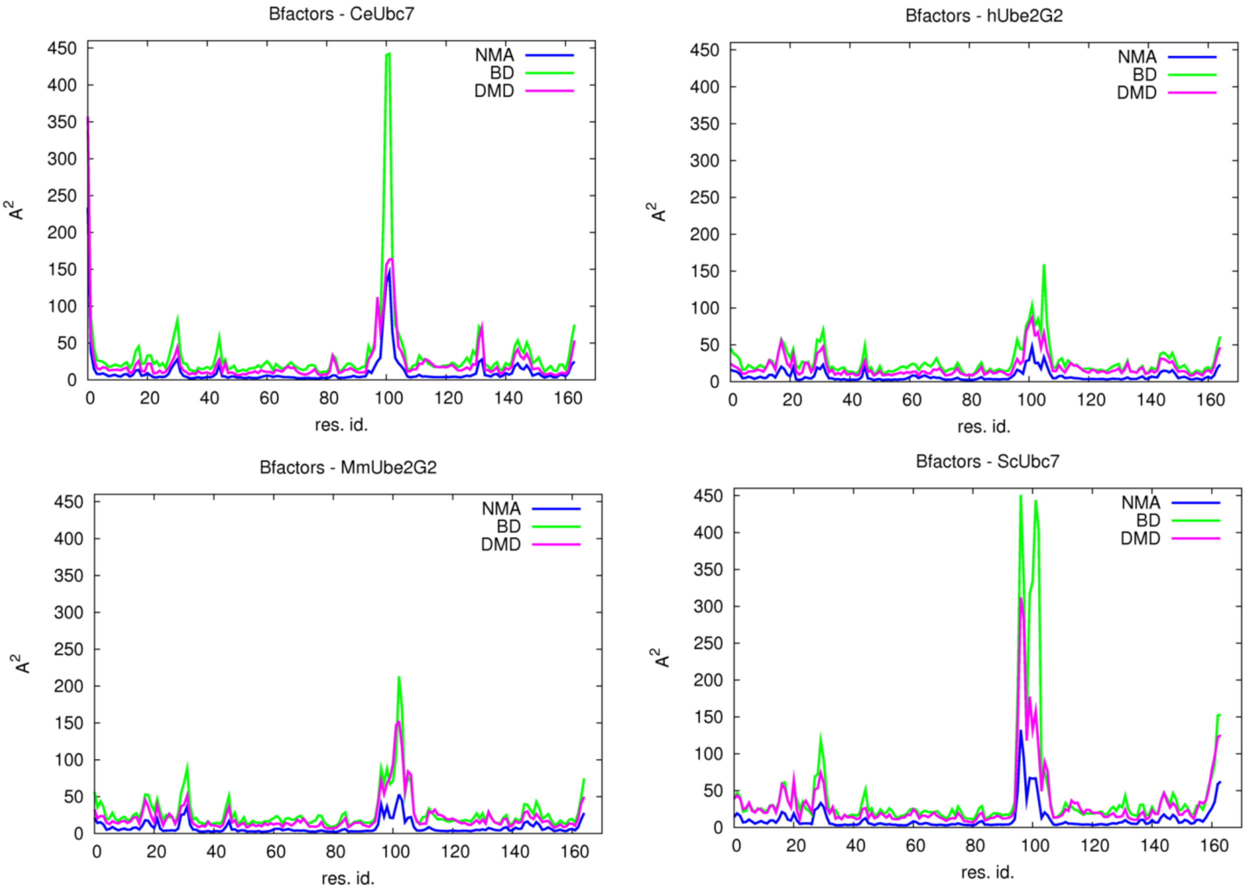

Figure 3. Rmsf profiles from MD simulations. The 3D structure of each E2 family 3 enzyme is used as a reference and each residue colored with different shade of colors according to Cα rmsf values calculated from the MD simulations (from light to dark colors for increasing rmsf values).

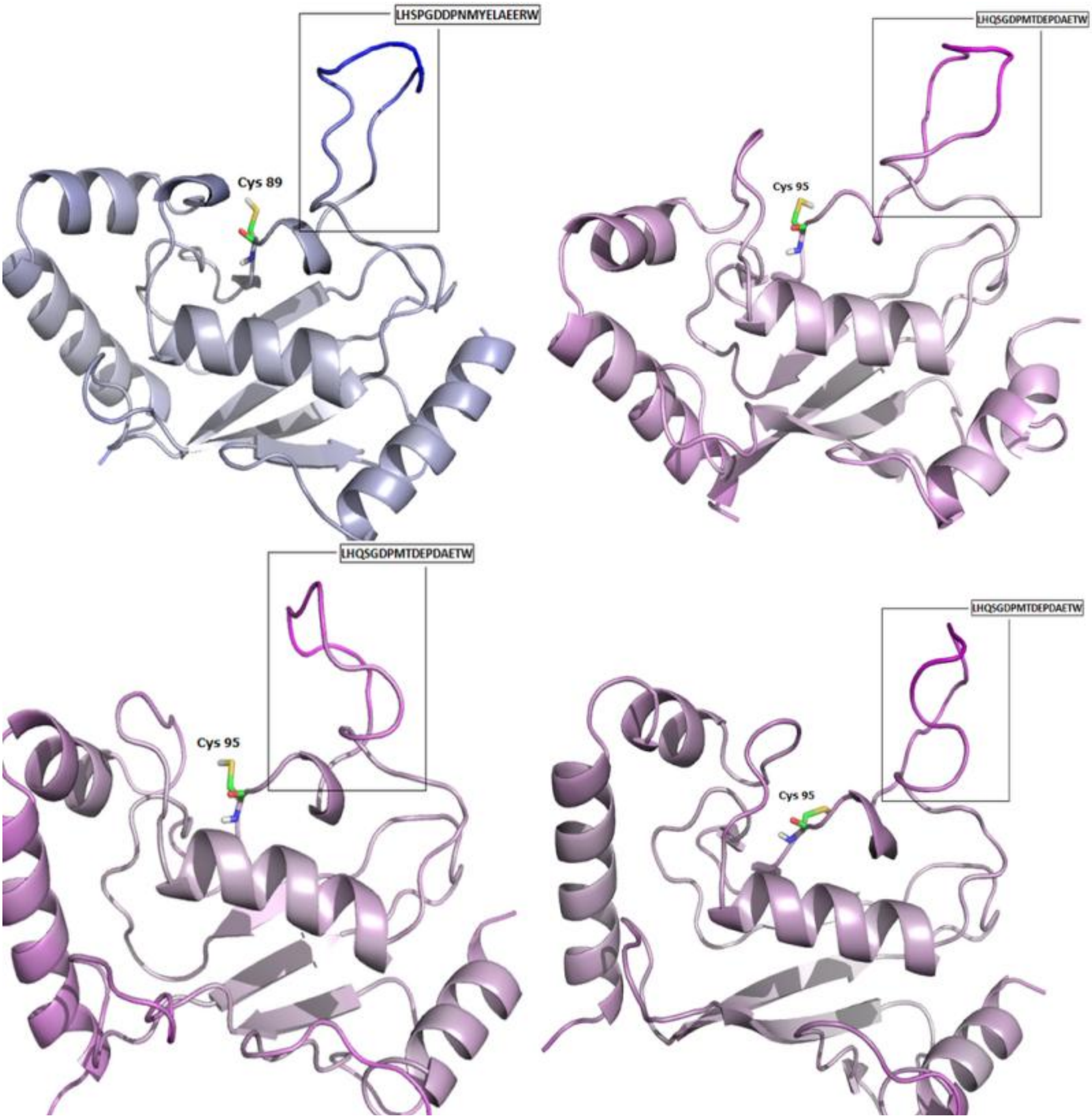

Text 1. Modeling of the E2-Ub complexes

Structural alignments have been performed with Dali in order to shed light on the mechanism of association between E2 enzymes and ubiquitin. The following known structures were used: Ubc1-Ub (pdb code: 1FXT), Ubc2D2-Ub (3A33), Ube2L6-Ub (2KJH).

These structures are complexes resulting from the covalent association between a molecule of ubiquitin and an E2 enzyme. As shown in the Fig.S4, ubiquitin in the known complexes is located in three different positions and orientations.

Since it has been experimentally verified (Ye et al., 2009) that the binding sites of E1 and E3 enzymes are partially overlapping on the structure of the E2s, it does not seem to be possible for ubiquitination to be a concerted process, but instead, it seems more plausible for it to be a pathway constituted by sequential steps. Thus, the structure of the complex E2-Ub is strongly influenced by the interactions between the E2 and the E1, since ubiquitin is trapped between these two enzymes during the process of transthiolesterification. For these reasons, a research based exclusively on the complexes E2-Ub would not be comprehensive of all of the elements taking part in this process.

The structures of the E1-E2-Ub complexes which we used for structural superimpositions in this work are 2NVU (UBA3-Nedd8-Ubc12) (Huang et al., 2007) and 2PX9 (SAE2-Ubc9) (Wang et al., 2007). In the first structure there are two NEDD8 molecules, one located at the transthiolation site and the other bound to the adenylation site of the E1 (UBA3). The E2 (Ubc12) in the complex is shown to interact non covalently with the E1 and the NEDD8 molecule at the transthiolation site. An interaction between helix 1 of the E2 and the UFD (Ubiquitin Fold Domain) of the E1 has been reported by Schulman et al. (2009): the electrostatic complementarity between these two components is crucial for the interaction between the two enzymes, since the introduction of mutations in helix 1 leads to a drastic loss of Ubl transfer activity. In order to resolve the structure and crystallize the entire complex, the catalytic cysteine of the E2 has been mutated into Ala to avoid the progression of the reaction. According to the authors, this mutation is the reason why the distance between the Ubc12 catalytic cysteine and NEDD8 Gly 76 is 30 A (fig S6A): they suppose that a subsequent rotation of the UFD domain interacting with Ubc12 would enable the ubiquitin transfer.

The E1 catalytic subunit (SAE2) without the regulatory subunit (SAE1) and SUMO has been crystallized and reported in Protein Data Bank with code 2PX9. It can be noticed in Fig. S4A-S5A that the E2 binding orientation is different from the one adopted by Ubc12 in the structure 2NVU. This could be due to the fact that the structure 2PX9 has been resolved in the absence of the regulatory subunit SAE1, SUMO and the UFD domain: in this context, the E2 might have established interactions with the E1 without the restraints determined by the presence of other molecules. Therefore, it is not possible to conclusively decide which one of these two structures (2PX9 and 2NVU) describes more precisely the interface between E1 and E2 enzymes. In light of this, we performed structural superimpositions having as templates both 2NVU and 2PX9: for each system three models have been produced, in which the E2s (from 1FXT, 3A33 and 2KJH) are superposed to the E2s of the respective system. The relative positions of ubiquitin in the models have been evaluated: as it can be noticed in Fig.S4-S5, the only orientation that satisfies the spatial restraints imposed by the E1's structure is the one that appears in 1FXT E2-Ub complex for both considered systems.

Finally, we superposed Ubc1 from 1FXT to the E2 from 2PX9 (Ubc9), which, in turn, had been previously superposed to 3CMM (Uba1), in oder to obtain a preliminary model of the complex Uba1-Ubc1-Ub from *S. cerevisiae* (Fig.S6).

Methods for model production: Homology models of the complexes *Sc*Cdc34-Ub have been created using as template structures Ubc1-Ub (pdb code: 1FXT), Ubc2D2-Ub (3A33), Ube2L6-Ub (2KJH). The software used for homology modeling procedure is Modeller version 9.9, and the models have been evaluated according to the energetic esteem of the structures performed by Modeller (DOPE score and molpdf).

ScCdc34 sequence has been extracted from Swissprot entry P14682, and the alignment with the template sequence Ubc1 is reported in supplementary materials.

Figure 4. Ubiquitin position and orientation is shown to be different in the three known complexes with E2 enzymes (PDB code 1FXT, 3A33, 2KJH).

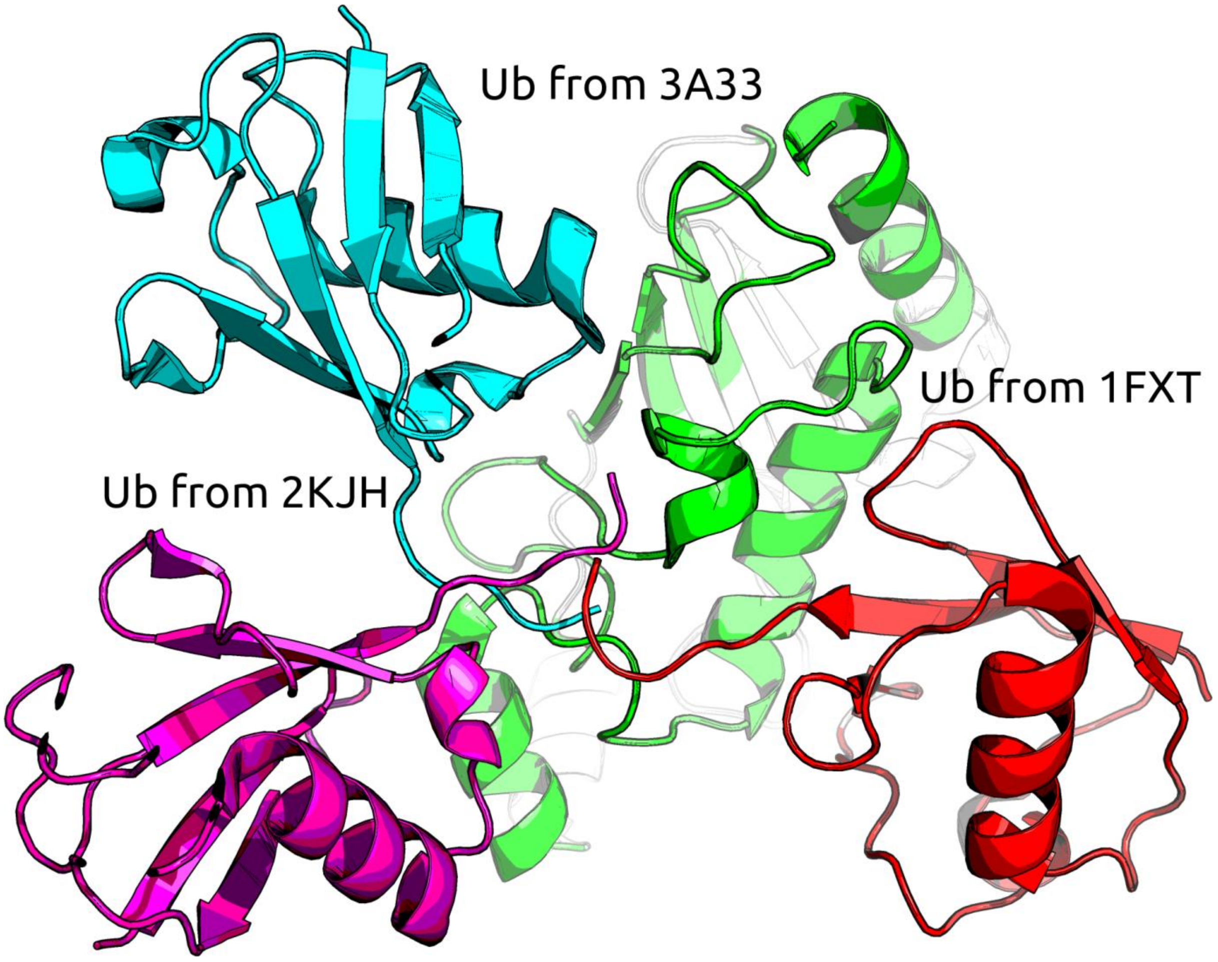

Figure 5. (A) E1 catalytic subunit SAE2 and Ubc9 have been crystallized in a structure with PDB code 2PX9 and it is the E1 structurally more similar to the Uba1 E1 enzymes for Ub. (B/C/D) Different ubiquitin positions have been obtained from the three known E2-Ub complexes through structural superimpositions. The only orientation that satisfies the spatial restraints imposed by the E1 structure is the one that appears in 1FXT E2-Ub complex (B).

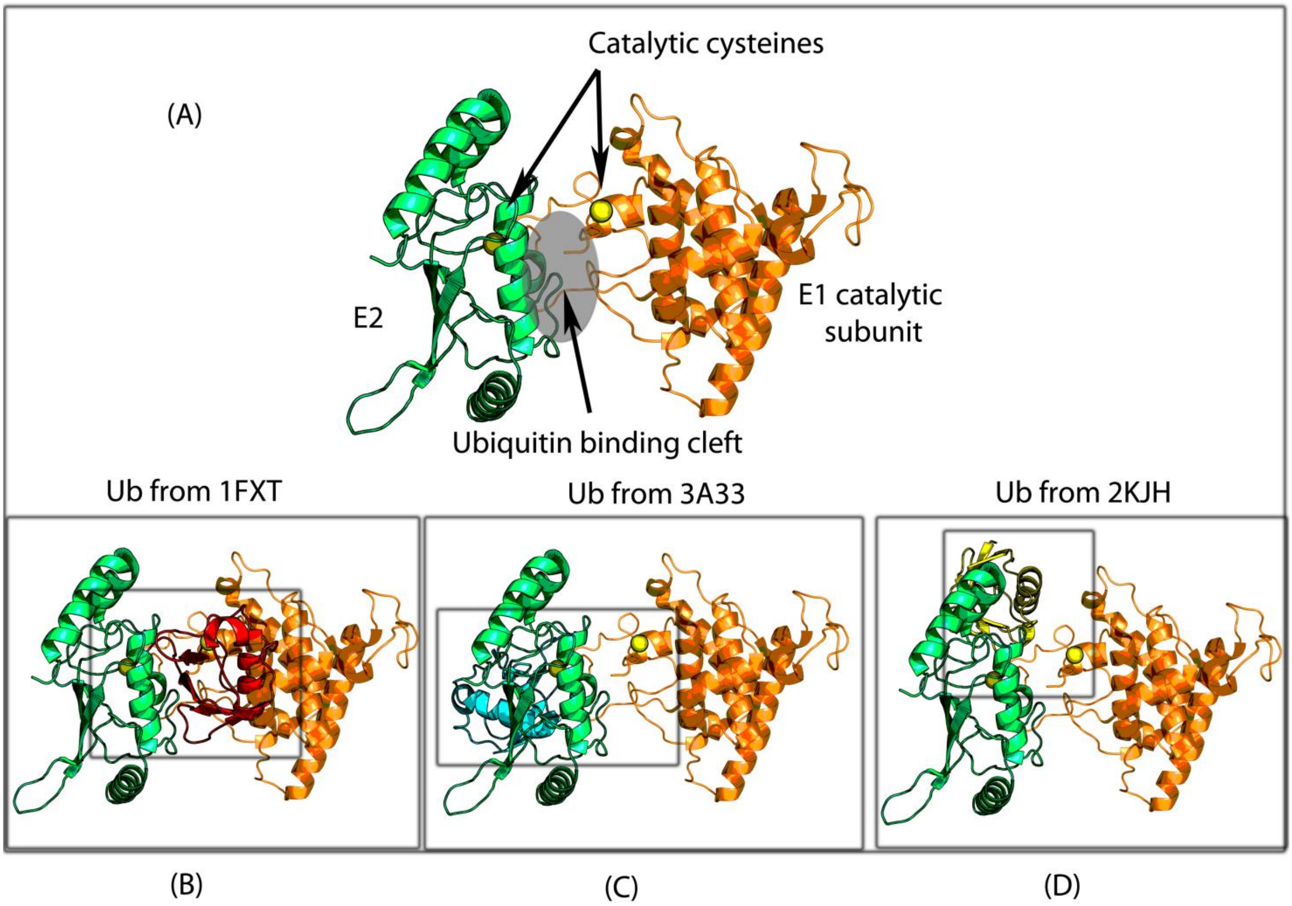

Figure 6. (A) UBA3 (E1) and Ubc12 have been crystallized in a structure with PDB code 2NVU. (B/C/D) Different ubiquitin positions have been obtained from the three known E2-Ub complexes through structural superimpositions. The only orientation that satisfies the spatial restraints imposed by the E1 structure is the one that appears in 1FXT E2-Ub complex (B).

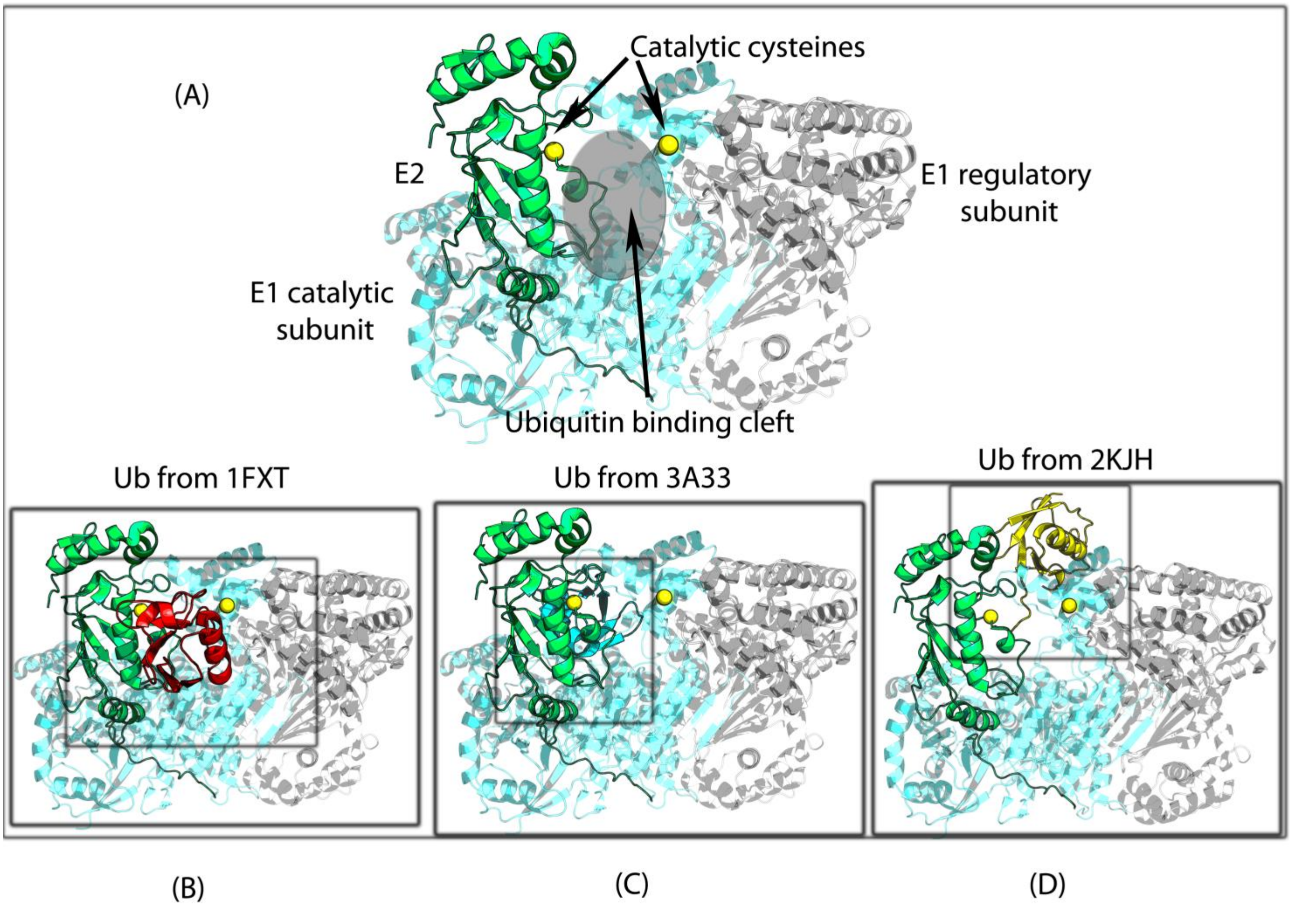

Figure 7. A preliminary model of an E1-E2 (family 3)-Ub complex has been obtained by superposing UBA1 structure (PDB code: 3CMM) to SAE2 structure (PDB code: 2PX9), using ubiquitin position as it appears in the complex Ubc1-Ub (1FXT). The catalytic cysteine of the E2 enzyme is indicated as yellow sphere and the E1 catalytic cysteine as yellow stick. Acidic L7 and phospho-S130 residues are shown as red spheres and the positively charged residues of Ub C-terminal chain as blue spheres.

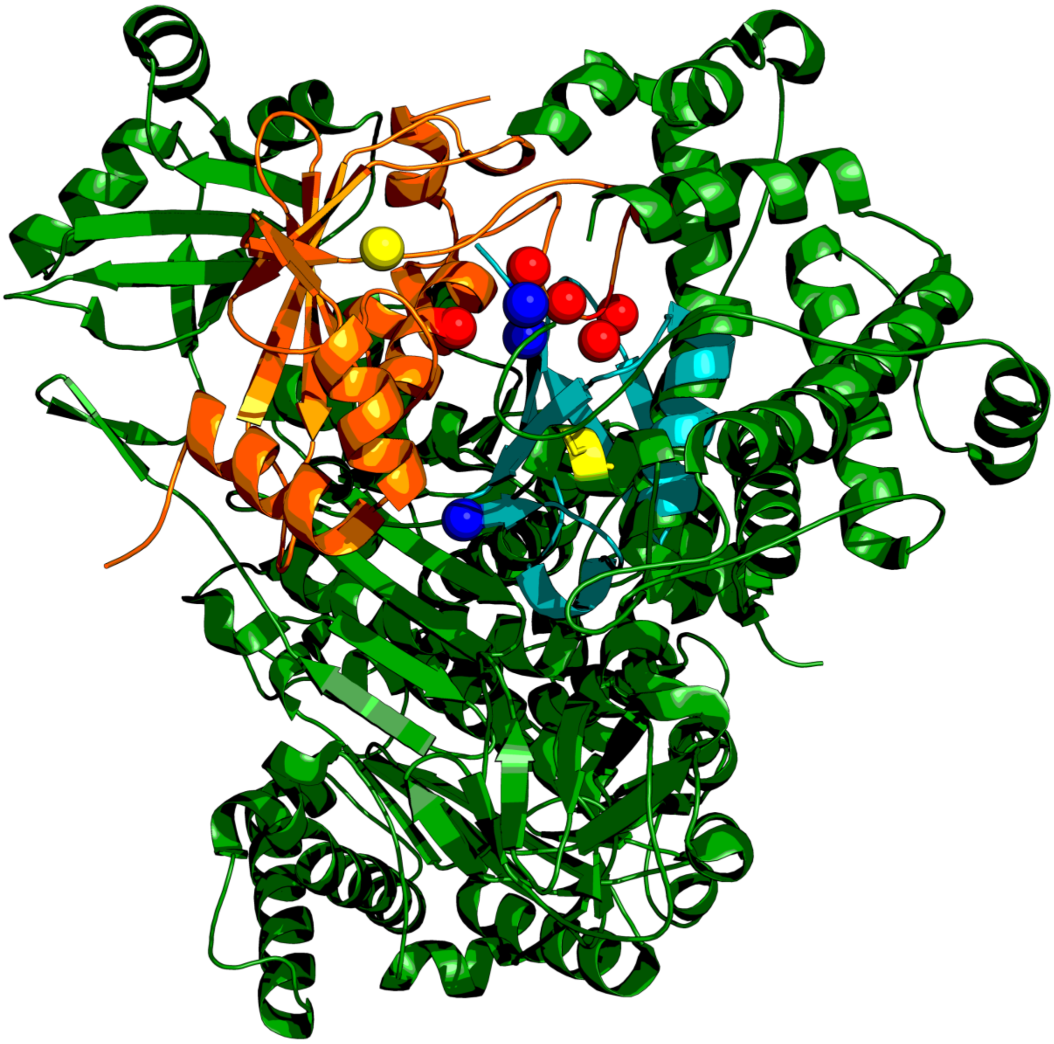

Figure 8. Mainchain Rmsd values over the simulations time of the all-atom MD trajectories. The Rmsd values have been calculated discarding the residues related to the acidic loop, due to its high flexibility, to better assess the convergence of the simulations.

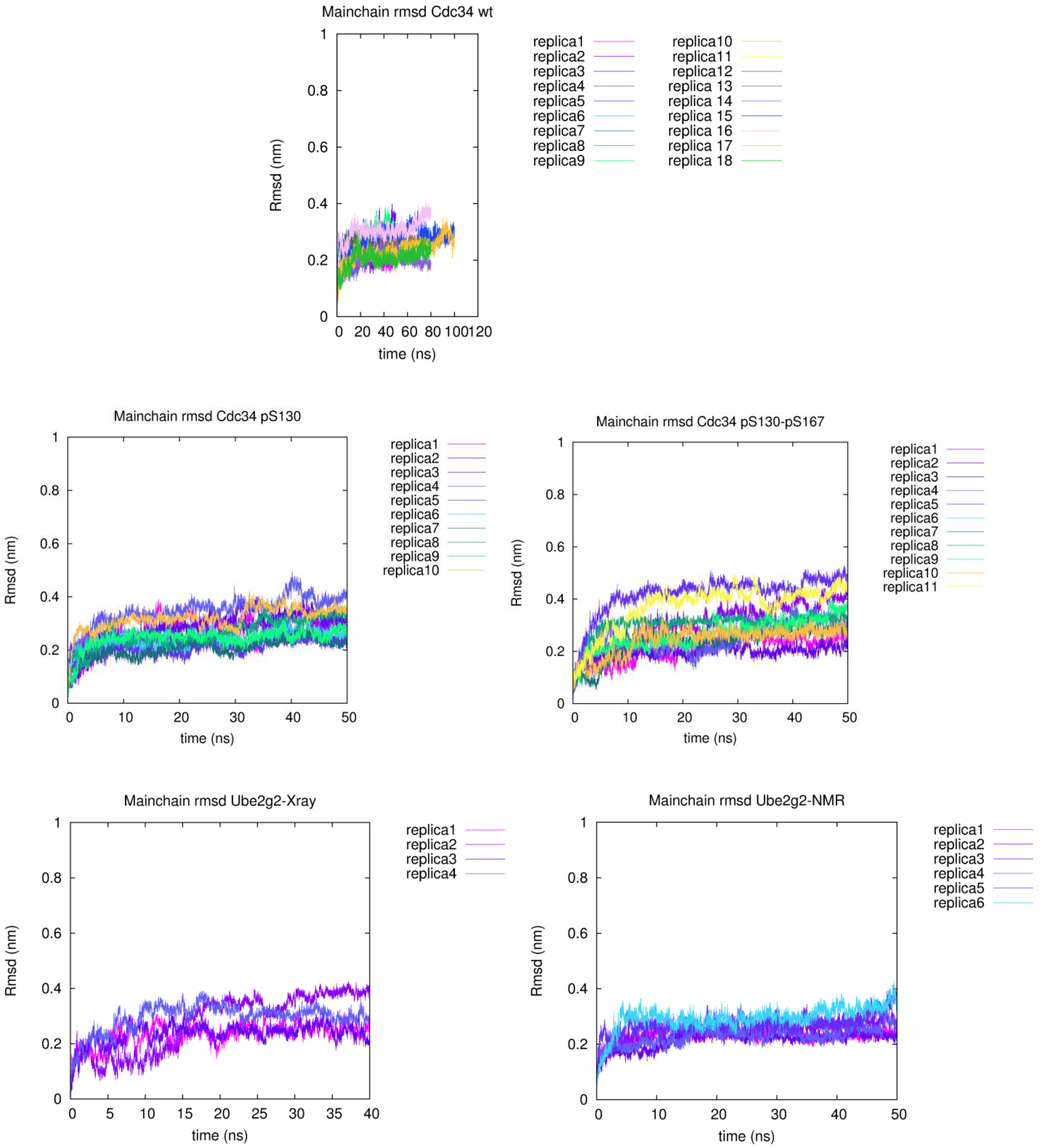

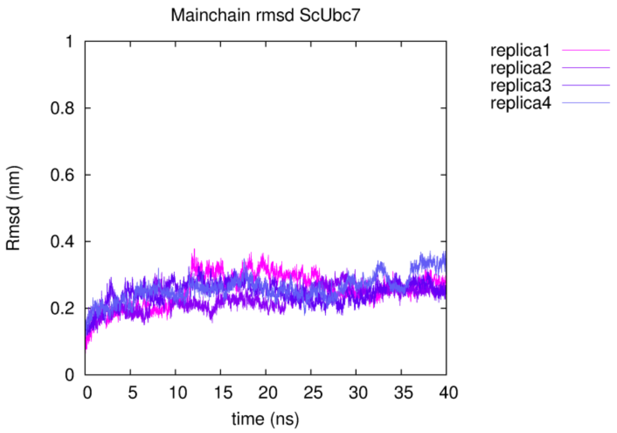
Mainchain rmsd ScUbc7
Rmsd (nm)
time (ns)
replica1
replica2
replica3
replica4
0
0.2
0.4
0.6
0.8
1
0
5
10
15
20
25
30
35
40

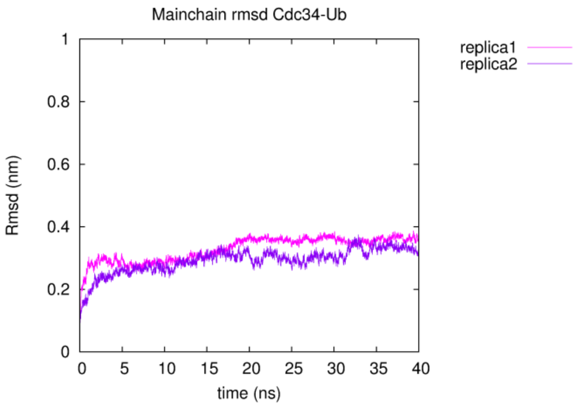
Mainchain rmsd Cdc34-Ub
Rmsd (nm)
time (ns)
replica1
replica2
0
0.2
0.4
0.6
0.8
1
0
5
10
15
20
25
30
35
40
